# Nickel-Resistance Determinants in *Acidiphilium* sp. PM Identified by Genome-Wide Functional Screening

**DOI:** 10.1371/journal.pone.0095041

**Published:** 2014-04-16

**Authors:** Patxi San Martin-Uriz, Salvador Mirete, Pedro J. Alcolea, Manuel J. Gomez, Ricardo Amils, Jose E. Gonzalez-Pastor

**Affiliations:** 1 Centro de Astrobiología (INTA-CSIC), Instituto Nacional de Técnica Aeroespacial, Torrejón de Ardoz, Madrid, Spain; 2 Centro de Investigaciones Biológicas (CSIC), Consejo Superior de Investigaciones Científicas, Madrid, Spain; 3 Centro de Biología Molecular Severo Ochoa (UAM-CSIC), Universidad Autónoma de Madrid, Madrid, Spain; University of Cambridge, United Kingdom

## Abstract

*Acidiphilium* spp. are conspicuous dwellers of acidic, metal-rich environments. Indeed, they are among the most metal-resistant organisms; yet little is known about the mechanisms behind the metal tolerance in this genus. *Acidiphilium* sp. PM is an environmental isolate from Rio Tinto, an acidic, metal-laden river located in southwestern Spain. The characterization of its metal resistance revealed a remarkable ability to tolerate high Ni concentrations. Here we report the screening of a genomic library of *Acidiphilium* sp. PM to identify genes involved in Ni resistance. This approach revealed seven different genes conferring Ni resistance to *E. coli*, two of which form an operon encoding the ATP-dependent protease HslVU (ClpQY). This protease was found to enhance resistance to both Ni and Co in *E. coli*, a function not previously reported. Other Ni-resistance determinants include genes involved in lipopolysaccharide biosynthesis and the synthesis of branched amino acids. The diversity of molecular functions of the genes recovered in the screening suggests that Ni resistance in *Acidiphilium* sp. PM probably relies on different molecular mechanisms.

## Introduction

Heavy metals are double-edged swords for life. While nanomolar concentrations afford organisms catalytic versatility and enable electron transfer reactions [1], [2], metal concentrations in the micromolar to millimolar range pose a threat to most life forms [3]. Nickel (Ni) is one of many heavy metals exhibiting this duality. To date, eleven enzymes have been reported to depend on Ni for their catalytic activity including urease, [Ni-Fe] hydrogenase, methyl-coenzyme M reductase, carbon monoxide dehydrogenase, acetyl-CoA synthase/decarbonylase (reviewed in [4]) or, more recently, the glycerol-1-phosphate dehydrogenase AraM from *Bacillus subtilis* [5]. Several of these enzymes are key for the colonization of highly selective niches such as the human gastric mucosa by the pathogen *Helicobacter pylori*, anaerobic environments by methanogens, or for the population of CO_2_- or CO-rich environments by acetogenic bacteria [6]. Yet, the presence of less than a part per mil of Ni is lethal to most microorganisms. For instance, the growth of *Saccharomyces cerevisiae* and *Escherichia coli*, is inhibited by 0.5 mM and 2 mM Ni, respectively [7], [8].

Four mechanisms have been proposed to explain Ni toxicity: i) replacement of the active metal in metalloproteins, ii) binding to catalytic residues of non-metalloenzymes, iii) allosteric inhibition of enzymes, and iv) induction of oxidative stress [9]. To tackle Ni toxicity, microorganisms have developed diverse mechanisms including extracellular detoxification, intracellular sequestration, modification of cation transport systems and active transport by efflux pumps [10], [11]. Of these mechanisms, inducible operon-encoded, energy-dependent specific efflux systems have been the most extensively studied. Examples of these Ni efflux systems are CnrCBA and NccCBA from the metal-resistant *Cupriavidus metallidurans* CH34 [12], [13], [14], the CznCBA efflux system of the pathogen *H. pylori* [15] and the *ncrABCY* determinant of the acidophilic *Leptospirillum ferriphilum* UBK03 [16], [17].

The identification of these heavy-metal resistance determinants is of great interest to the bioleaching industry. The use of microorganisms for the extraction of metals from low-grade ores can make profitable an otherwise economically unviable process [18]. The success of this procedure, however, depends largely on the ability of these microorganisms to thrive in heaps where one or more heavy-metals can reach toxic concentrations [18], [19]. Bacterial adaptation to such extreme environments is not well understood and predicting a bacterial maximum metal resistance for bioleaching purposes is yet unachievable. Thus, identifying resistance determinants from natural metal resisters could contribute to the understanding of metal tolerances in bioleaching microorganisms. Pioneering studies in the study of metal resistance in *Acidiphilium* have focused in plasmid-mediated resistance to arsenic in *Acidiphilium multivorum* AIU301 [20], [21]. The number of metal-resistance determinants identified in acidophilic species continues to grow, especially with the advent of new approaches, such as metagenomics [8].

In this study, we identified genes conferring nickel resistance to *Acidiphilium* sp. PM (DSM 24941), an *alphaproteobacterium* isolated from the surface waters of a 6 m-deep dam in the initial course of Rio Tinto [22] (sampling point RT8 according to nomenclature used in [23]). Rio Tinto is a naturally acidic, heavy metal-rich river located in southwestern Spain [24]. Earlier studies performed in RT8 have reported a water pH of 2.5, a conductivity of 6.63 mS cm^−1^ and high concentrations of heavy metals (Fe, 33.6 mM; Zn, 1.6 mM; Mn, 1.1 mM; Cu, 0.3 mM; Ni, 17 µM [23]). These conditions favour the growth of *Acidiphilium* species, which dominate both in the water column and in the sediments of this part of the river [23]. *Acidiphilium* sp. PM has been previously studied for its ability to transfer electrons to graphite electrodes [25] and to mediate in the formation of iron carbonate precipitates [26]. Moreover, its genome was recently sequenced, annotated and its central metabolism was partially reconstructed [22], which provided a valuable tool.

The characterization of the heavy metal resistance in *Acidiphilium* sp. PM revealed a remarkable ability to tolerate high nickel concentrations. To uncover the genes responsible for Ni resistance a genomic library of *Acidiphilium* sp. PM was constructed and screened for Ni-resistant (Ni^r^) clones. Seven open reading frames (ORFs) involved in nickel resistance were retrieved, two of which form an operon encoding the protease HslVU (ClpQY). This protease was found to enhance resistance to both Ni and Co in *E. coli*.

## Materials and Methods

### Bacterial strains, media and growth conditions

*Acidiphilium* sp. PM was grown aerobically at 30°C in media containing (per liter): (NH_4_)_2_SO_4_, 2 g; KCl, 0.1 g; K_2_HPO_4_ · 3H_2_O, 0.327 g; MgSO_4_ · 7H_2_O, 0.25 g and Ca(NO_3_)_2_ · 4H_2_O, 0.1 g. pH was adjusted to 2.5 with H_2_SO_4_, then autoclaved and supplemented with filter-sterilized glucose (100x stock solution: 200 g l^−1^), modified Wolfe's minerals solution (1000x stock solution) per liter: nitrilotriacetic acid, 1.5 g; MgSO_4_ · 7H_2_O, 3.0 g; MnSO_4_ · 2H_2_O, 0.5 g; NaCl, 1.0 g; FeSO_4_ · 7H_2_O, 0.1 g; CoSO_4_ · 7H_2_O, 0.18 g; NaSeO_3_ · 5H_2_O, 0.3 g; ZnSO_4_ · 7H_2_O, 0.18 g; CuSO_4_ · 5H_2_O, 0.01 g; KAl(SO_4_)_2_ · 12H_2_O, 0.02 g; H_3_BO_3_, 0.01 g; Na_2_MoO_4_ · 2H_2_O, 0.01 g and NiCl_2_ · 6H_2_O, 0.025 g) and modified Wolfe’s vitamin solution (1000x stock solution (per liter): biotin, 20 mg; pyridoxine-HCl, 10 mg; riboflavin, 50 mg; calcium D-pantothenate, 50 mg; 4–aminobenzoic acid, 50 mg; folic acid, 20 mg; thiamine-HCl, 50 mg; nicotinic acid, 50 mg and cobalamin, 50 mg). Wolfe’s mineral and vitamins solutions were described in [27]. To prepare plates, Wolfe’s vitamin and mineral solutions were replaced by yeast extract, 0.1 g l^−1^. Agar was autoclaved separately, cooled down to 50°C and added to a final concentration of 10 g l^−1^.

The characterization of the resistance of *Acidiphilium* sp. PM to heavy metals was carried out using Zn (II)-, Co (II)-, Cd (II)-, Cu (II)- and Ni (II)-sulphate salts. Solutions containing heavy metals were sterilized by filtration through 0.22 µm-pore filters. Growth in liquid cultures was monitored by measuring absorbance at 600nm (A_600nm_).

*E. coli* DH10B was grown aerobically in Terrific Broth modified (Sigma-Aldrich, St. Louis, MO) or LB media at 37°C. These media were supplemented with 50 µg ml^−1^ ampicillin (LB-Ap) to maintain pBluescript II SK^+^ plasmid (pSKII^+^) (Stratagene, La Jolla, CA). The selection of Tn5 insertion mutants was carried out in LB-Ap plates supplemented with 50 µg ml^−1^ kanamycin (LB-Ap-Kan).

Assessing the metal resistance of the *E. coli* clones required drop assays in which serial dilutions of overnight-grown cultures were plated onto LB-Ap plates supplemented with 2.25 mM NiSO_4_ · 6H_2_0, 0.8 mM CdSO_4_ · 8/3H_2_0, 1.5 mM ZnSO_4_ · 7H_2_0, 1.25 mM CoSO_4_ · 7H_2_0 or 4.5 mM CuSO_4_ · 5H_2_0. The concentrations above are the minimum inhibitory concentrations for *E. coli* DH10B (pSKII^+^). After inoculation, plates were incubated overnight at 37°C. *E. coli* DH10B (pSKII^+^) was used as a negative control in the experiments.

### Isolation of nucleic acids and construction of a shotgun genomic library of *Acidiphilium sp.* PM

DNA from *Acidiphilium* sp. PM was isolated using phenol-chloroform-isoamyl alcohol extraction [28], partially digested with Sau3AI (Roche Applied Science, Mannheim, Germany) and separated in a 10–40% sucrose gradient by isopycnic ultracentrifugation. Fragments with sizes ranging from 2 to 6 kb were ligated overnight to BamHI-digested, Shrimp Alkaline Phosphatase-dephosporilated pBluescript II SK^+^ vector using T4-DNA ligase (Roche). Purified ligation products were used to transform *E. coli* DH10B electrocompetent cells (Invitrogen, Carlsbad, CA). Electroporation was performed with a Micropulser Electroporation Apparatus (BioRad, Hercules, CA) following the manufacturer’s instructions. Plasmid preparations were carried out using QIAprep Spin Miniprep Kit (Qiagen). Nucleic acid concentrations were measured with an ND-1000 Spectrophotometer (NanoDrop Technologies Inc., Wilmington, DE).

### Screening for nickel-resistant genes

Approximately 12000 library clones were plated onto LB-Ap plates supplemented with 2.25 mM Ni (LB-Ap-Ni). After an overnight incubation, the colonies were considered as putative nickel-resistant (Ni^r^) clones. To exclude that mutations in the host chromosome were responsible for the resistance, recombinant plasmids from these Ni^r^ clones were isolated and transformed into new *E. coli* DH10B cells. Re-transformed cells were tested for Ni resistance on LB-Ap-Ni plates.

### In silico analysis of Ni-resistant clones

Inserts from the recombinant plasmids of Ni^r^ clones were sequenced on an Applied Biosystems 3730*xl* DNA Analyzer (Applied Biosystems, Foster City, CA) using M13pUC primers and BigDye Terminator v3.1 Cycle Sequencing Kit (Applied Biosystems). Sequence reads were then aligned against the *Acidiphilium* sp. PM draft genome (accession no. NZ_AFPR00000000) using BLASTN [29]. The output was processed with a custom Perl script that defined the boundaries of each cloned fragment by pairing forward and reverse sequence read matches that fulfilled the following conditions: (i) e-value for both alignments < 1e^−100^, (ii) convergent orientation of the mapped sequences and (iii) 10 kb maximum distance between the 5-prime ends of the mapped sequences.

The public annotation of the *Acidiphilium* sp. PM genome [22] was used to identify open reading frames (ORFs) overlapping with the cloned fragments. Genes overlapping at least 5% with the clone were considered in the analysis. Transmembrane domain analysis was performed using TMHMM v. 2.0 (http://www.cbs.dtu.dk/services/TMHMM/).

### Identification of Ni-resistant determinants

Cloned fragments that contained several ORFs were further analyzed by *in vitro* transposon mutagenesis and/or subcloning to determine the ORF(s) responsible for Ni resistance. *In vitro* transposon mutagenesis was performed using the EZ-Tn5 In-Frame Linker Insertion Kit (Epicentre, Madison, WI) according to the manufacturer’s instructions. Products of transposon insertion reactions were transformed in *E. coli* DH10B by electroporation and selected in LB-Ap-Kan plates. Approximately two hundred transformants from each reaction were first patched on LB-Ap-Kan plates, and then streaked on LB-Ap-Kan plates with 2.25 mM Ni. Ni-sensitive transformants were an indication that the interrupted ORF was involved in Ni resistance. The insertions sites were mapped by sequencing using transposon-specific primers. In addition, several Ni-resistant transformants had their insertions mapped to verify that the remaining ORFs were not involved in Ni-resistance.

Subcloning of ORFs was performed either by PCR amplification or by digestion and re-ligation of the recombinant plasmids. PCR amplification of *orf2-orf3* from pSRNi16 was performed in a final volume of 50 µl using the following reaction mixture: 250 ng of genomic DNA, 350 µM of each dNTP, 1U of Expand Long Template DNA polymerase (Roche), 10% DMSO, and 300 nM of forward (CAGGTTCTAGATCAACATATTCGTGACCTGATCG) and reverse primers (CAGGTAAGCTTGCCGGTTACTATAGGGTCAGGAC). Underlined nucleotides indicate restriction sites for XbaI and HindIII, respectively. Thermal cycling consisted of one single 3-min step at 94°C; 35 cycles of 1 min at 94°C, 1 min at 52.9°C, 4 min at 68°C; and a final 10-min elongation step at 68°C. PCR products were checked on agarose gels, then excised and purified using the QIAquick Gel Extraction kit (Qiagen). These amplicons were then digested with the appropriate restriction enzymes and ligated to equally-digested pSKII^+^ vector. To include their native expression sequences (promoters and ribosome binding sites), *ca.* 200-bp regions upstream of the start codon were also amplified. Subcloning of the different ORFs by digestion and re-ligation was performed using the appropriate restriction enzymes in each case (see Figure S2 in File S1). Eventually, all the recombinant plasmids generated were transformed into *E. coli* DH10B and tested for nickel resistance as explained above.

### Determination of cellular Ni concentration

*E. coli* DH10B (pSKII^+^) and the Ni^r^ clones were inoculated in LB broth containing 50 µg ml^−1^ ampicillin and incubated at 37°C on rotary shakers. In early stationary phase, Ni was added to a final concentration of 4 mM and cultures were incubated for one additional hour. Cells were then harvested by centrifugation and washed three times with ultrapure H_2_O before pellets were lyophilized, pulverized and dissolved in H_2_O:HCl:HNO_3_:H_2_O_2_ 3∶1∶4∶0.5 (v/v) by a closed vessel microwave digestion system. Ni content (expressed here as mg of Ni per g of dry weight) was determined by inductively coupled plasma-mass spectrometry (ICP-MS). Cellular Ni content is the sum of intracellular Ni and Ni covalently bound to the cell envelope.

### Heat shock resistance

*E. coli* DH10B carrying an empty pSKII^+^ or pSKII^+^ bearing *hslVU* was grown in LB broth supplemented with ampicillin (50 µg ml^−1^) until cultures reached early exponential phase (A_600nm_ = 0.3–0.4). At that point, 1 ml of each culture was centrifuged at 12000 g for 2 minutes. Cell pellets were then washed with Phosphate Buffered Saline (PBS) buffer (pH 7.4), centrifuged again and resuspended in 1 ml of the same buffer. Cells were then incubated at 50°C in a heating block. Aliquots were removed at 0 and 30 minutes. To determine viability at each time point, serial dilutions of the aliquots in PBS buffer were plated on LB agar and incubated overnight at 37°C. Percentage of survival was calculated as the number of colony forming units (cfu) ml^−1^ remaining after the temperature treatment divided by the cfu ml^−1^ at time zero. Each experiment was repeated at least three times.

### UV-radiation resistance

Cultures of *E. coli* DH10B carrying an empty pSKII^+^ or pSKII^+^ bearing *hslVU* were grown overnight in LB broth supplemented with ampicillin (50 µg ml^−1^). One ml of culture adjusted to A_600nm_ = 2 was centrifuged at 12000 g for 2 minutes. Cell pellets were then washed with PBS buffer (pH 7.4), centrifuged again and resuspended in 1 ml of the same buffer. Serial dilutions were plated onto LB agar and irradiated at room temperature with germicidal radiation (λ = 254 nm-lamp operating at 1.55 W m^−2^) for 0 and 5 seconds. Plates were then incubated overnight at 37°C. Percentage of survival was calculated as the number of cfu ml^−1^ remaining after exposure to UV-radiation divided by the cfu ml^−1^ in the non-exposed plates. Each experiment was repeated at least three times.

### Hydrogen peroxide resistance

*E. coli* DH10B carrying an empty pSKII^+^ or pSKII^+^ bearing *hslVU* was grown in LB broth supplemented with ampicillin (50 µg ml^−1^) until cultures reached early exponential phase (A_600 nm_ = 0.3–0.4). At that point, 1 ml aliquots were removed and incubated at 37°C in the presence of 2.5 mM H_2_O_2_. Aliquots were removed at 0 and 30 minutes. To determine viability, serial dilutions of the aliquots were plated on LB agar and incubated overnight at 37°C. Percentage of survival was calculated as the number of cfu ml^−1^ remaining after the treatment with H_2_O_2_ divided by the cfu ml^−1^ at time zero. Each experiment was repeated at least three times.

### Acid resistance

Acid resistance experiments were performed as described by Guazzaroni and co-workers [30]. Cultures of *E. coli* DH10B carrying an empty pSKII^+^ or pSKII^+^ bearing *hslVU* were grown overnight in LB broth supplemented with ampicillin (50 µg ml^−1^). One microliter of these cultures was transferred to 1 ml of PBS (pH 7.2) and to 1 ml of LB broth (pH 1.8). Cells in LB broth were then incubated in a heating block at 37°C for 60 min. Cells in PBS were used to determine initial cell populations whereas cells in LB broth were used to calculate final cell populations. The cfu ml^−1^ for both PBS and LB broth were calculated by plating serial dilutions onto LB agar plates which were then incubated overnight at 37°C. Percentage of survival was calculated as the number of cfu ml^−1^ remaining after the acid treatment divided by the initial cfu ml^−1^ at time zero. Each experiment was repeated at least three times.

### Phylogenetic analysis of HslVU

16S rRNA gene sequences of 14 acidophilic and 14 non-acidophilic taxa were retrieved from GenBank and their phylogenetic affiliations were determined using the ARB software package (http://www.arb-home.de/) [31] and SILVA database (release 102) [32]. The alignment was performed with ARB FAST ALIGNER and then exported to Mega 5 software package [33] applying a bacterial positional variability filter (pos_var_Bacteria_102). A phylogenetic tree was constructed with the Neighbor-Joining method using the genetic distances calculated with the Maximum Composite Likelihood method. In parallel, the amino acid sequences of HslV and HslU of the same organisms were retrieved from GenBank and concatenated. An alignment was generated with ClustalW [34] using Mega 5. A phylogenetic tree was inferred by using the Maximum Likelihood method based on the Jones–Taylor–Thornton (JTT) amino acid substitution model. In both phylogenetic trees, the branch support was assessed with a bootstrap analysis of 1000 replicates.

### Nucleotide sequence accession numbers

The nucleotide sequences obtained in this work have been deposited in the GenBank database (http://www.ncbi.nlm.nih.gov/genbank/) under the accession numbers listed in Table 1.

**Table 1:** Description of the ORFs contained in the recombinant plasmids of the nickel-resistant clones.

Clone (insert size)	GC content (%)	GenBank accession no.	ORF	Length (aa)	Proteins in *Acidiphilium* sp. PM using blastn (aa length), GenBank accession no. of the protein	Protein coverage (missing end)^1^	Function	TMH in the clone (TMH in the entire protein)^4^
pSRNi5 (4233 bp)	69	KC110840	1	75	Hypothetical protein (145), EGO96502	51% (C-term)	Unknown	2 (4)
			**2**	**183**	**ATP-dependent protease HsIV (183), EGO96503**	**100%**	**ATP-dependent protease HslVU**	**0 (0)**
			**3**	**439**	**ATP-dependent protease ATP-binding subunit HslU (439), EGO96504**	**100%**	**ATP-dependent protease HslVU**	**0 (0)**
			4	114	Hypothetical protein (114), EGO96505	100%	Unknown	2 (2)
			5	473	Amidase (473), EGO96506	100%	Hydrolysis of amides	0 (0)
pSRNi6 (4106 bp)	70	KC110841	1	209	3-oxoacyl-(acyl-carrier-protein) reductase (249), EGO95637	84% (C-term)	Dissociated (type II) fatty acid biosynthesis system	0 (0)
			**2**	**319**	**Malonyl CoA-acyl carrier protein transacylase (319), EGO95636**	**100%**	**Fatty-acid biosynthesis in bacteria**	**0 (0)**
			**3**	**196**	**Polysaccharide export protein (196), EGO95635**	**100%**	**Polysaccharide export**	**1 (1)**
			**4**	**551**	**Non-specific protein-tyrosine kinase (647), EGO95634**	**77% (C-term)** ^2^	**Exopolysaccharide biosynthesis**	**0 (0)**
pSRNi16 (2281 bp)	69	KC110843	**1**	**537**	**Dihydroxy-acid dehydratase (617), EGO95524**	**87% (C-term)**	**Fourth step in the biosynthesis of Ile and Val**	**0 (0)**
			2	68	Hypothetical protein (68), EGO95523	100%	Unknown	0 (0)
			3	128	RND efflux transporter (107), EGO95522	20% (N-term)^3^	Involved in hopanoid biosynthesis	4 (12)
pSRNi20 (2728 bp)	63	KC110844	**1**	**889**	**Glycosyl transferase, Group 1, EGO96186**	**81% (N-term)**	**Cell envelope biogenesis, outer membrane**	**0 (0)**

ORFs involved in Ni resistance are shown in bold type. (1) C-term and N-term represent carboxy and amino ends, respectively. (2) The amino acid sequence of non-specific protein-tyrosine kinase deposited in GenBank (EGO95634) is incomplete on the carboxy end. The full protein, as derived from A. *cryptum* JF5 (ABQ30731) and A. *multivorum* AIU301 (BAJ80915) consists of 718 residues. (3) The amino acid sequence of RND efflux transporter deposited in Genbank (EGO95522) is incomplete on the amino end. The full protein, as derived from A. *cryptum* JF5 (ABQ30260) and A. *multivorum* AIU301 (BAJ80194) consists of 858 residues. (4) TMH stands for transmembrane helixes.

## Results and Discussion

### Metal resistance in *Acidiphilium sp.* PM

**Figure 1:**
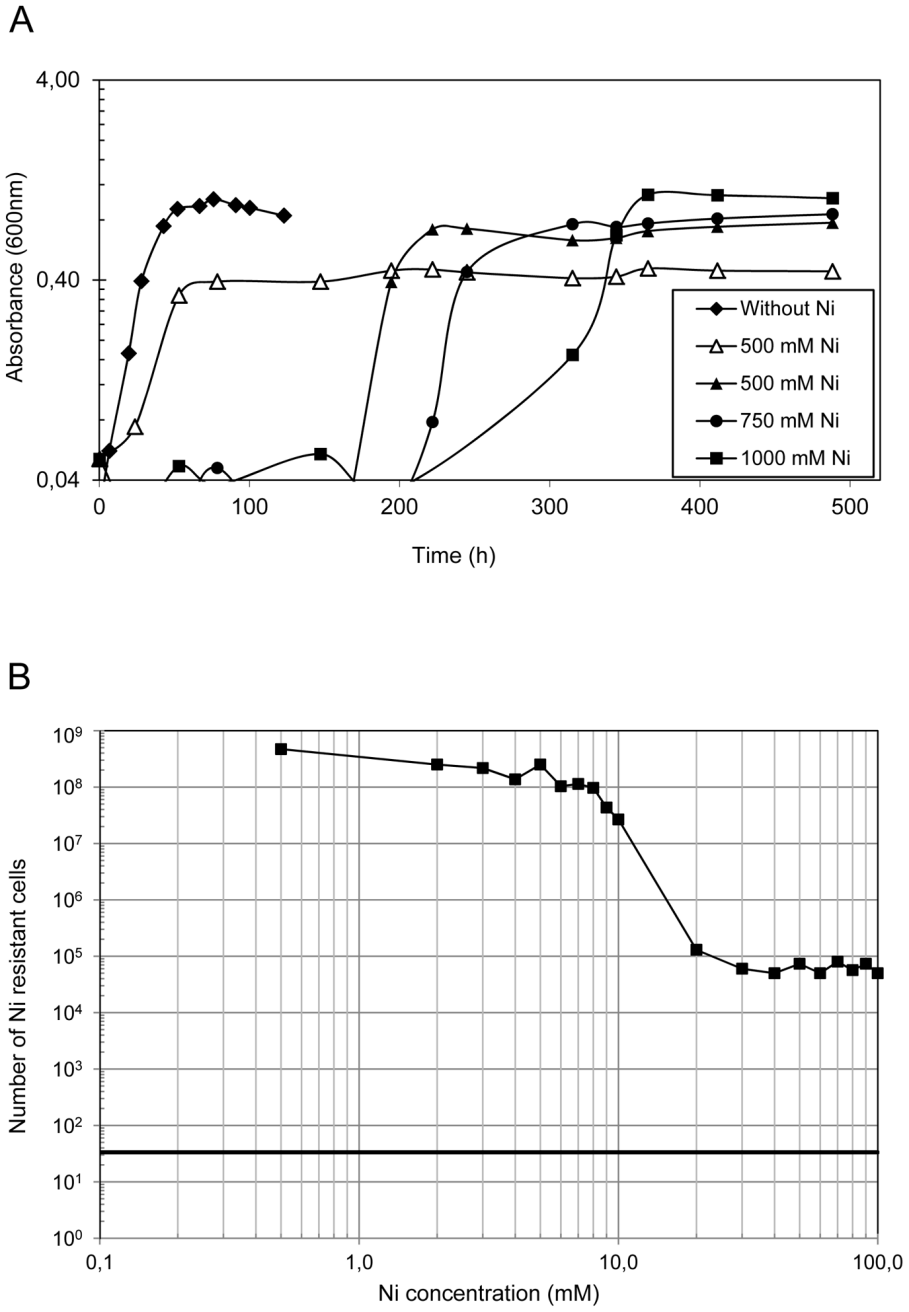
Growth of *Acidiphilium* sp. PM in the presence of Ni. A) Growth of *Acidiphilium* PM in the presence of Ni, with (empty symbols) or without (black symbols) pre-growth in media containing 500 mM. B) Viability of *Acidiphilium* sp. PM in the presence of increasing concentrations of Ni.

*Acidiphilium* sp. PM is an *alphaproteobacterium* isolated from the heavy-metal laden waters of Rio Tinto (southwestern Spain) [22]. The characterization of its heavy metal resistance revealed an outstanding capacity to grow in the presence of high nickel concentrations (up to 1 M). Interestingly, this extreme tolerance to Ni did not require preadaptation to lower concentrations of the metal and consistently emerged in unexposed populations. Growth in high Ni concentrations was preceded by long lag phases (often in excess of ten days), which were greatly reduced when cells were pre-cultured in Ni-containing media (Fig. 1A).

Drop assays performed in Ni-containing plates revealed that most cells are sensitive to 20 mM Ni and that only a fraction of the population is capable of growing at concentrations up to 100 mM Ni (the highest concentration tested in plates) (Fig. 1B). Interestingly, Ni-resistant (Ni^r^) colonies presented increasingly longer lag phases in plates containing larger Ni concentrations.

This extreme Ni resistance was consistently observed on multiple cultures from single-picked colonies, which suggests that *Acidiphilium* sp. PM has the ability to rapidly evolve an extreme Ni^r^ phenotype. Paradoxically, Ni concentration throughout Rio Tinto remains below 1 mM [23].

This tolerance to Ni is among the highest reported for any prokaryote [19], [35]. For this reason, we aimed to identify the genes involved in Ni resistance. To this end, a genomic library of *Acidiphilium* PM was constructed and screened for Ni^r^ clones.

### Construction of a genomic library and screening for Ni^r^ clones

A shotgun genomic library was constructed in *E. coli* DH10B using the high-copy-number plasmid pBluescript II SK^+^ (pSKII^+^). Around 10^5^ recombinants were obtained with an average insert size of 2.2 kb (range 0.5 to 7 kb).

**Figure 2:**
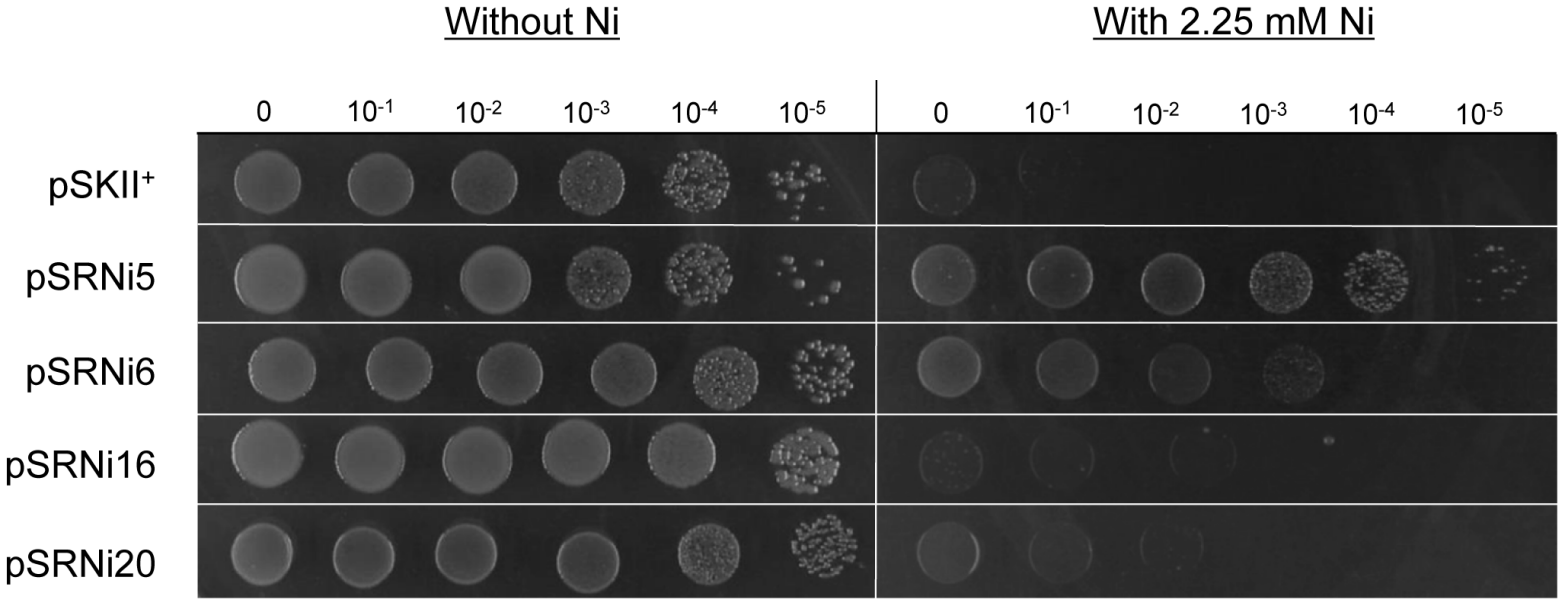
Ni resistance of the clones rescued in the screening of the genomic library. Serial dilutions of overnight-grown cultures were plated on LB-Ap plates with (right) and without 2.25 mM Ni (left). Asssays were performed in triplicate using independent cultures.

To make sure that a representative portion of the genome was screened, roughly 12000 recombinant clones were plated onto LB-Ap plates containing 2.25 mM Ni (a lethal concentration for *E. coli* DH10B). These 12000 clones contained *ca.* 26 Mb of cloned DNA (equivalent to 6.5 times the size of *Acidiphilium* sp. PM genome (3.98 Mb) [22]). After an overnight incubation at 37°C, several Ni^r^ colonies were recovered. To exclude the possibility that chromosomal mutations in the host were responsible for the resistant phenotype, recombinant plasmids from the Ni^r^ clones were extracted and transformed again into *E. coli* DH10B. Re-transformed clones were tested for Ni resistance using drop assays. All clones (including the control) grew to similar cell densities in the absence of Ni (Fig. 2 left). Therefore, the different growths observed in the presence of Ni can only be attributed to the genes encoded in the recombinant plasmids (Fig. 2 right). Clones carrying pSRNi5 and pSRNi6 exhibited the highest levels of Ni resistance.

Earlier works in our laboratory showed that Ni resistance determinants may also confer resistance to Co and Cd [8]. For this reason all four Ni^r^ clones were tested for cross-resistance to Co(II), Cd(II), Cu(II) and Zn(II) (Fig. S1 in File S1). Transformants carrying pSRNi6 were found to tolerate 0.8 mM Cd(II) and all clones except pSRNi20 resisted at least 1.25 mM Co(II). On the other hand, none of the clones presented significantly higher resistance to Cu or Zn than the control (Fig. S1 in File S1). Similar Ni-Co cross-resistance has been reported previously for other Ni-resistance determinants [8], [13], [36], [37].

### Cellular Ni content

Some metal resistance mechanisms prevent the accumulation of nickel through active transport efflux pumps or by extracellular chelation of the metal. Others tend to favour the intracellular accumulation by sequestering Ni in histidine-rich vacuoles [10], [11]. To gather information on the mechanism of Ni resistance in our Ni^r^ clones, the cellular Ni content of the clones was measured.

**Figure 3:**
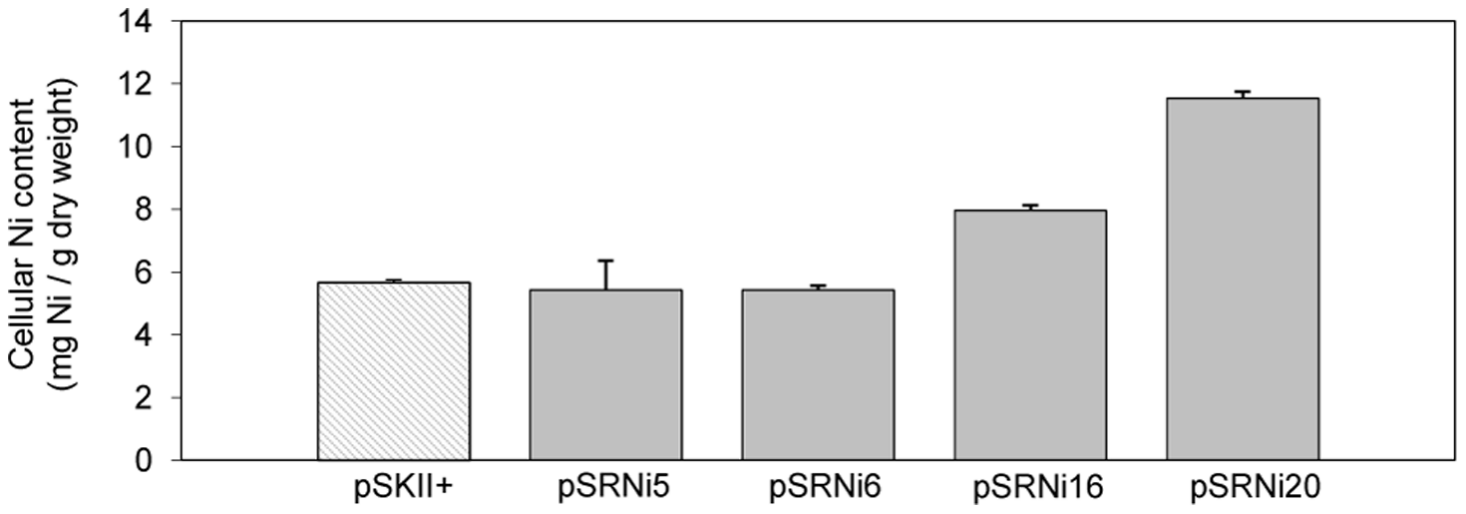
Cellular Ni concentration of the Ni-resistant clones. Concentrations were measured after growing for 1 hour in the presence of 4

Cells in early stationary phase were supplemented with 4 mM Ni and incubated for another hour. After harvesting the cells, the intracellular Ni content of the clones was measured using inductively coupled plasma-mass spectrometry (ICP-MS). As shown in Fig. 3 cells bearing pSRNi20 seemed to accumulate a substantially larger amount of cellular Ni compared to control cells (bearing an empty pSKII^+^). On the other hand, no clones seemed to actively export Ni outside the cell.

### Identifying the genes conferring Ni resistance

**Figure 4:**
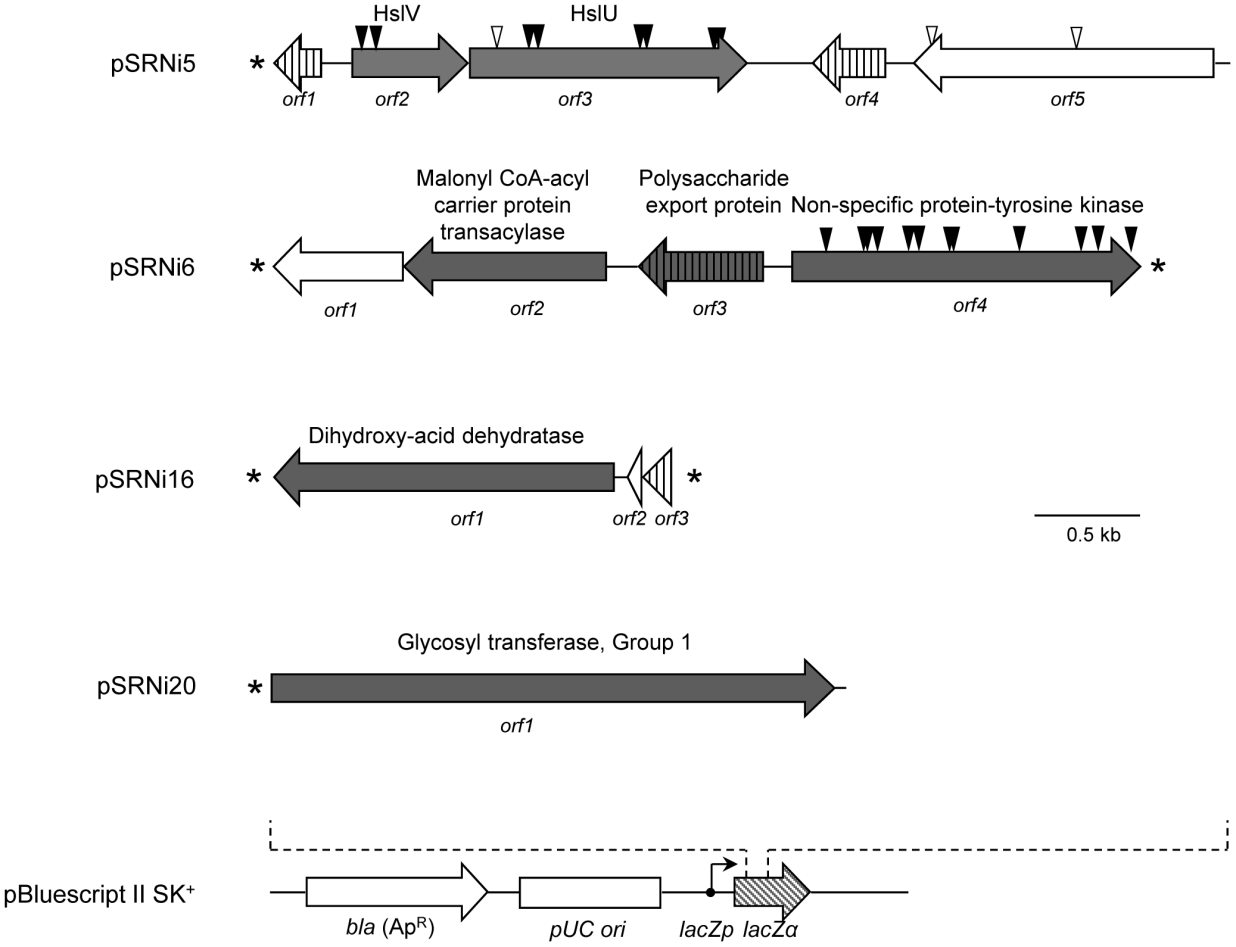
Genetic organization of the recombinant plasmids that confer Ni resistance to *E. coli*. ORFs involved in Ni resistance are shown in grey. ORFs with predicted transmembrane helices are shaded with vertical bars. Asterisks indicate incomplete ORFs. Vertical arrowheads indicate transposon insertions that either abolish the resistant phenotype (filled in black) or do not affect the resistant phenotype (empty arrowheads). The map of the vector (pBluescript II SK+) is represented at the bottom.

In order to identify the ORFs conferring Ni resistance in each recombinant plasmid, inserts were end-sequenced and mapped against the draft genome of *Acidiphilium* sp. PM. Its genome comprises the chromosome and 9 plasmids with sizes 650, 270, 190, 90, 70, 50, 20–30, 4.8 and 3 kb (as determined by gel electrophoresis). Surprisingly, all of the insert sequences aligned with the chromosome and not with the plasmids. In fact, earlier reports on *Acidiphilium* resistance determinants for arsenic [21], cadmium and zinc [38] revealed they were plasmid-encoded. Insert sequences had a GC-content ranging between 63% and 70%, comparable to the overall 68% GC-content of *Acidiphilium* sp. PM [22]. The gene organization, protein identification and transmembrane domain predictions of the ORFs contained in the cloned DNA fragments are summarized in Fig. 4 and Table 1.

Five ORFs were identified in pSRNi5, four in pSRNi6, three in pSRNi16, and a single ORF was found in pSRNi20 (Fig. 4). The identification of the gene(s) responsible for Ni resistance was accomplished by subcloning and/or *in vitro* transposon mutagenesis in the recombinant plasmids harbouring two or more ORFs. Plasmid pSRNi20 contained a single ORF encoding a glycosyl transferase; therefore, we concluded that differences in Ni resistance between this clone and the control (empty vector) were necessarily caused by that single ORF.

The glycosyl transferase encoded in plasmid pSRNi20 belongs to the GT-B fold type of glycosyltransferases. It contains several conserved domains including a domain found in GT1 family of glycotransferases and two conserved RfaG domains. RfaG domains were named after protein RfaG of *E. coli*, an enzyme that catalyzes the addition of the first glucose to the core lipopolysaccharide (LPS), which forms part of the outer membrane [39], [40]. It is likely that the glycosyl transferase in pSRNi20 also participates in the biosynthesis of the LPS, hence contributing to an enhanced permeability barrier that helps maintain Ni ions outside the cell. Besides, there is evidence that sugars, including fructose, maltose and rhamnose, can coordinate Ni^2+^ ions [41]. An enlarged LPS would therefore allow the formation of higher numbers of LPS-Ni^2+^ complexes, preventing Ni free ions from entering the cell. This would also explain the increased levels of cellular Ni observed in this clone.

In the case of pSRNi6, the interruption of the non-specific protein-tyrosine kinase by transposon mutagenesis abolished the nickel resistant phenotype. This protein contains several conserved domains (GumC, Wzz, eps_fam), all of which are conserved in proteins participating in exopolysaccharide or lipopolysaccharide biosynthesis. On the other hand, no insertion mutants were recovered that interrupted any of the three other ORFs present in pSRNi6 (Fig. 4). Therefore, to discard their role in Ni resistance, a fragment that comprised *orf1-orf2-orf3* was subcloned. The resulting plasmid conferred a Ni-sensitive phenotype. Furthermore, plasmids which included only the kinase gene or a combination of the kinase and the polysaccharide export protein genes conferred only slight resistance to Ni (Fig. S2B in File S1). Overall, these results suggest that while the non-specific protein tyrosine kinase contributes to Ni resistance, the presence of at least the malonyl CoA-acyl carrier protein transacylase and the polysaccharide export protein is also necessary to explain the high levels of resistance to Ni observed in the clone bearing pSRNi6. Interestingly, the polysaccharide export protein contains a predicted transmembrane helix and is very likely embedded in the membrane, which supports its involvement in the biosynthesis of the lipopolysaccharide. As in the case of pSRNi20, we suggest that the overexpression of genes involved in the biosynthesis of components of the cell envelope might create a denser extracellular barrier that prevents Ni ions from entering the cell. The disparities in the cellular Ni content of pSRNi6 and pSRNi20 could be ascribed to the different Ni binding properties of the components of the cell envelope each clone produces.

In pSRNi16, the Ni-resistance determinant was identified by subcloning the ORFs. Subcloning *orf1*, which encodes a putative dihydroxy-acid dehydratase, yielded cells with a resistance to Ni higher than those carrying the intact pSRNi16 (Fig. S2C in File S1). Additionally, subcloning both *orf2* and the complete *orf3* together in the direction of the *lacZ* promoter (*lacZp*) yielded a Ni-sensitive clone (Fig. S2C in File S1). Overall, these data indicated that the dihydroxy-acid dehydratase was the ORF responsible for the Ni^r^ phenotype observed in pSRNi16-bearing clones. Dihydroxy-acid dehydratases (EC 4.2.1.9) catalyze the third step in the biosynthesis of the branched amino acids valine, leucine and isoleucine [42]. Increased branched amino acid concentrations in response to both acid and metal stress have been previously reported [43], [44] and might explain the increased Ni resistance observed in *E. coli* transformed with pSRNi16. Moreover, dihydroxyacid dehydratases contain 4Fe-4S clusters in their active site, which are particularly susceptible to superoxide generated by Ni [45], [46], [47]. Thus, the overexpression of the dihydroxy acid dehydratase from *Acidiphilium* sp. PM could compensate the loss of function of *E. coli* enzyme in the presence of Ni.

Plasmid pSRNi5 carries an insert encoding 5 ORFs. Transposon mutagenesis of pSRNi5 yielded eight Ni-sensitive mutants with insertions in *orf2* and *orf3* (Fig. 4). On the other hand, two insertions in *orf5* (encoding an amidase) did not affect the resistant phenotype (Fig. 4). o*rf2* and *orf3* encode HslV and HslU proteins, which form an operon-encoded protease named HslVU (also known as ClpQY). The involvement of HslVU in the resistance to Ni was confirmed by subcloning *hslVU* (Fig. S2A in File S1). Indeed, the subcloned operon conferred the same level of resistance as the complete pSRNi5. On the other hand, the subcloning of the amidase yielded a Ni-sensitive clone (Fig. S2A in File S1). Overall, this data indicated that the *hslVU* operon was solely responsible for the resistance observed in the pSRNi5-bearing clone. The Ni cellular content of the transformants carrying pSRNi5 or an empty plasmid pSKII^+^ is similar, which indicates a possible intracellular protection, such as the recycling of mis-folded (hence unfunctional) proteins. Similarly to other caseinolytic proteases (Clp), HslVU is composed of an AAA+ ATPase responsible for the unfolding and protein recognition (HslU or ClpY) and a small peptidase (HslV or ClpQ) [48], [49], [50]. Together with Lon, ClpXP and ClpAP, HslVU is responsible for 70 to 80% of the protein degradation *in vivo* [51]. Proteases are responsible for the degradation of mis-folded proteins and those proteins no longer needed by the cell, which is crucial in adaptation to stress. The role of protein degradation in the response to metals has been reported in the past. In the presence of high intracellular Zn concentrations, *E. coli* Zn response regulator ZntR binds both Zn and DNA, activating ZntA (an ATPase essential for Zn export). However, when Zn concentrations are low, ZntR does not bind Zn or DNA, which makes it more unstable and susceptible to degradation by proteases ClpXP and Lon [52]. Moreover, protease complex HslVU is considered to be the bacterial homolog of the proteasome [50], the main complex for protein degradation in eukaryotes and archaea. A study by Forzani and co-workers [7] showed that the expression of the maize proteasome α subunit conferred resistance to Ni, Co and Cd in yeast. Similarly, we have observed that the proteasome homolog HslVU confers resistance to both Ni and Co but not to Cd, Cu or Zn (Fig. S3 in File S1).

### Effect of the overexpression of protease HslVU under other stress conditions

**Figure 5:**
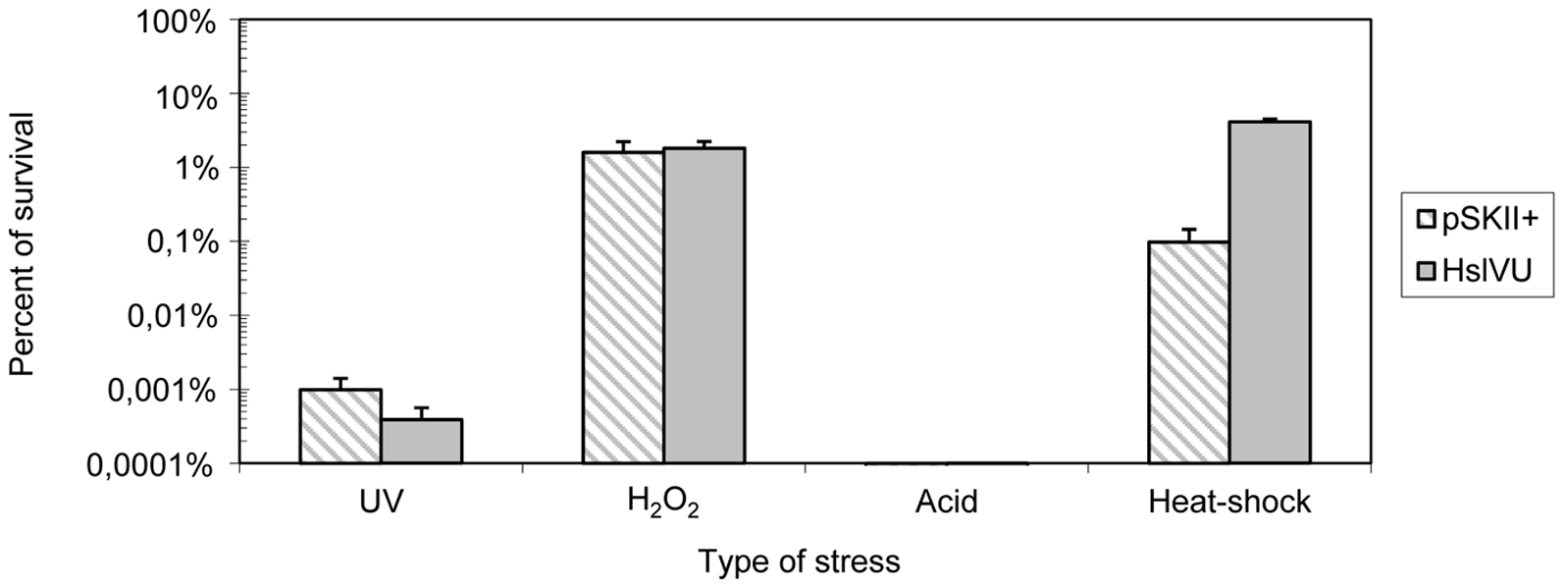
Effect of the overexpression of protease HslVU on *E. coli* survival under different stresses. Percentage of survival of *E.coli* DH10B bearing empty pSKII^+^ vector (barred columns) or pSKII^+^ carrying operon *hslVU* (solid columns). Cells were exposed to the following stresses: 5-seconds of germicidal UV light, 30 minutes in the presence of 2.5 mM H_2_O_2_, 1 hour at pH 1.8 or 30-minute incubations at 50°C. Percentage of survival was calculated as the number of colony forming units (cfu) ml^−1^ remaining after the treatment divided by the number of cfu ml^−1^ at time zero. In all the experiments, *E. coli* DH10B carrying the empty vector (pSKII^+^) was used as the negative control.

Protease HslVU plays an important role in the heat shock response by controlling the *in vivo* turnover of both the heat shock sigma factor (σ^32^) and abnormal proteins in *E. coli* [53]. At the same time, the transcription of *hslVU* is under the regulation of σ^32^ [54]. To test whether the overexpression of HslVU from *Acidiphilium* could confer greater resistance to heat shock in *E. coli*, cells carrying *hslVU* were grown to early exponential phase and exposed to 50°C. After a 30 minute exposure, cells overexpressing HslVU had survival rates 42 times greater than the control carrying an empty vector (pSKII^+^: 0.10±0.05; HslVU: 4.11±0.37) (Fig. 5). This is in agreement with earlier reports which showed an increased transcription of operon *hslVU* upon heat shock induction [54].

Interestingly, HslVU also plays a regulatory role in the response to oxidative stress by participating in the degradation of SulA, a cell division inhibitor activated in the SOS response in *E. coli* [55], [56]. To test whether overexpression of HslVU from *Acidiphilium* sp. PM could also confer resistance to oxidative stress in *E. coli*, the subclone bearing *hslVU* was exposed to UV radiation (λ = 254 nm) and to hydrogen peroxide. After a five-second exposure to UV radiation, cells overexpressing HslVU did not show enhanced survival compared to control cells (Fig. 5). Similarly, no differences were observed between the control and the HslVU-bearing clone when cells were exposed to 2.5 mM H_2_O_2_ (Fig. 5). Although H_2_O_2_ is known to trigger SOS response, overexpression of HslVU did not increase *E. coli* resistance to H_2_O_2_, probably because hydrogen peroxide causes further damages to the cell (*e.g.* lipid peroxidation) than can be repaired by the SOS response.

It has been reported that acidic conditions can lower intracellular pH, causing protein unfolding and uncoupling of oxidative phosphorylation [57], [58], [59]. This damage can eventually lead to cell death. It has been recently described that the heterologous expression of *Terriglobus saanensis* protease ClpXP dramatically increases the survival of *E. coli* at pH 1.8 [30]. To test whether HslVU could also enable the growth of *E. coli* in acidic conditions, the clone carrying *hslVU* was exposed to pH 1.8 for one hour. No differences in the percentage of survival were observed between the *hslVU*-bearing clone and the control (Fig. 5). This suggests that its contribution to the turnover of proteins is insufficient to maintain cell growth under acidic conditions.

### Phylogenetic analysis of HslVU

Microorganisms that share the same niche tend to exchange genes that are useful in adaptation to stress or changing conditions. A recent study suggested that the acidophilic red alga *Galdieria sulphuraria* could have acquired up to 5% of its genome from various bacteria and archaea through horizontal gene transfer (HGT) [60]. Genes acquired by this red alga included some involved in heavy metal detoxification. The fact that *Acidiphilium* sp. PM carries a plasmid that is 91% identical to *Acidithiobacillus ferrooxidans* plasmid pTF4.1 [22], indicates that gene exchange might have also taken place in Rio Tinto.

The finding that the expression of HslVU enhances growth under different types of stress (presence of Ni and Co and heat shock), led us to explore the possibility that it had been acquired through HGT. A comparative analysis was performed using both acidic and non-acidic taxa. Representatives of the acidic species included the three main genera found in Rio Tinto (*Acidithiobacillus* spp., *Leptospirillum* spp. and *Acidiphilium* spp.) as well as other species found in acidic environments.

Phylogenetic trees were built using 16S rRNA gene sequences and a concatenated amino acid sequence of HslV and HslU subunits. In both trees, sequences belonging to *Acidiphilium* spp. clustered together in a separate group from those of *Leptospirillum, Acidithiobacillus* and the rest of acidic species. The similarity in the topologies of HslVU-based and 16S rRNA-based trees, suggests that operon *hslVU* of *Acidiphilium* was acquired through vertical transmission rather than horizontal transfer among the species tested (Fig. S4A and Fig. S4B in File S1).

### Further prospects

This is the first attempt to identify the genes involved in Ni resistance in *Acidiphilium*, one of the most conspicuous dwellers of acidic environments and a natural metal resister. This screening revealed seven different genes that confer Ni resistance to *E. coli*. Our future goal is to individually test the relevance of these genes in the resistance to Ni of *Acidiphilium* sp. PM. Attempts to conjugate or transform *Acidiphilium* sp. PM (in order to construct mutants) have been so far unsuccessful. Hence, further work will focus on the development of genetic tools for the manipulation of this bacterium. In addition, our recent reports suggest that several chaperones and proteases from acidophiles could be involved in resistance to specific environmental stresses: ClpB in the resistance to As[61], ClpXP in the resistance to acidic pH [30] and HslVU (ClpQY) in resistance to Ni (this work). The fact that these proteins could have evolved to provide resistance to different extreme conditions in acidophiles, has led us to start their characterization.

## Supporting Information

File S1 **Figure S1.** Determination of heavy-metal cross-resistance of the four nickel-resistant clones. Serial dilutions of overnight-grown cultures were plated on LB-Ap plates containing 0.8 mM Cd, 1.25 mM Co, 4.5 mM Cu, or 1.5 mM Zn. **Figure S2.** Identification of the *orfs* involved in Ni resistance by subcloning. ORFs from the environmental DNA inserts of pSRNi5 (A), pSRNi6 (B) and pSRNi16 (C) were subcloned and tested for Ni resistance. Serial dilutions of overnight cultures were plated in LB-Ap plates containing 2.25 mM Ni. Assays were performed in triplicate using independent cultures. ORFs involved in Ni resistance are shown in grey. ORFs with predicted transmembrane helices are shaded with vertical bars. Asterisks indicate incomplete ORFs. pSRNi5_*orf2*: ATP-dependent protease hsIV; pSRNi5_*orf3*: ATP-dependent protease ATP-binding subunit HslU; pSRNi5_*orf5*: amidase; pSRNi6_*orf*1: 3-oxoacyl-(acyl-carrier-protein) reductase; pSRNi6_*orf2*: malonyl CoA-acyl carrier protein transacylase; pSRNi6_*orf3*: polysaccharide export protein; pSRNi6_*orf4*: non-specific protein-tyrosine kinase; pSRNi16_*orf1*: dihydroxy-acid dehydratase; pSRNi16_*orf2*: hypothetical protein; pSRNi16_*orf3*: RND efflux transporter. **Figure S3.** Determination of heavy-metal cross-resistance of protease HslVU (*orf2-orf3*). Serial dilutions of overnight-grown cultures were plated on LB-Ap plates containing 0.8 mM Cd, 1.25 mM Co, 4.5 mM Cu, or 1.5 mM Zn. **Figure S4.** Exploring possible horizontal gene transfer of operon *hslVU* in acidic environments. Phylogenetic trees of 14 acidic and 14 non-acidic species are shown as reconstructed from the 16S rRNA gene (A), and the concatenated amino acid sequences of HslV and HslU (B). Sequences belonging to *Acidiphilium* sp. PM are shown in bold text and those of acidic species are denoted by asterisks. Bootstrap values are indicated at the nodes. Scale bars correspond to 5% (A) or 10% (B) sequence divergence. The amino acid sequences for HslV and HslU used in the analysis are the following: *Rhodospirillum rubrum* F11, AEO50144 and AEO50145; *Escherichia coli* str. K-12 substr. DH10B, ACB04944 and ACB04943; *Pseudomonas aeruginosa* PAO1, AAG08438.1 and AAG08439; *Bacillus subtilis* subsp. *subtilis* str. 168, CAB13488 and CAB13489; *Salinibacter ruber* M8, CBH24765 and CBH24767; *Ralstonia solanacearum* GMI1000, CAD13571 and CAD13570; *Borrelia burgdorferi* N40, ADQ29063 and ADQ29174; *Thermotoga maritima* MSB8, AAD35606 and AAD35607; *Planctomyces brasiliensis* DSM 5305, ADY60041 and ADY60040; *Acidithiobacillus ferrooxidans* ATCC 23270, ACK80450 and ACK79019; *Acidithiobacillus ferrooxidans* ATCC 53993, ACH84568 and ACH84569; *Acidithiobacillus ferrivorans* SS3, AEM46768 and AEM46767; *Acidithiobacillus caldus* SM-1, AEK57130 and AEK57129; *Acidithiobacillus caldus* ATCC 51756, EET27426 and EET27425; *Acidiphilium multivorum* AIU301, BAJ80804 and BAJ80803; *Acidiphilium* sp. PM, EGO96503 and EGO96504; *Acidiphilium cryptum* JF-5, ABQ30620 and ABQ30619; *Leptospirillum rubarum*, EAY57767 and EAY57766; *Leptospirillum* sp. Group II '5-way CG', EDZ39580 and EDZ39579; *Acidocella* sp. MX-AZ02, EKM99984 and EKM99983; *Acetobacter pasteurianus* IFO 3283-01, BAH99172 and BAH99173; *Gluconacetobacter diazotrophicus* PAl 5, ACI52812 and ACI52813; *Gluconobacter oxydans* H24, AFW01689 and AFW01690; *Roseomonas cervicalis* ATCC 49957, EFH12145 and EFH12146; *Azospirillum lipoferum* 4B, CBS88212 and CBS88211; *Thiomonas intermedia* K12, ADG31586 and ADG31587; *Sulfobacillus acidophilus* DSM 10332, AEW05868 and AEW05867; *Alicyclobacillus acidocaldarius* subsp. *acidocaldarius* DSM 446, ACV58411 and ACV58412; *Leptothrix cholodnii* SP-6, ACB36091 and ACB36092.
(DOCX)

## References

[1] Gray H, Stiefel EI, Valentine JS, Bertini I (2006) Biological Inorganic Chemistry: Structure and Reactivity: University Science Books.

[2] Messerschmidt A, Huber R, Wieghart K, Poulos T (2005) Handbook of Metalloproteins: Wiley.

[3] Haferburg G, Kothe E (2007) Microbes and metals: interactions in the environment. J Basic Microbiol 47: 453–467. 18072246 10.1002/jobm.200700275

[4] Mulrooney SB, Hausinger RP (2003) Nickel uptake and utilization by microorganisms. FEMS Microbiol Rev 27: 239–261. 12829270 10.1016/S0168-6445(03)00042-1

[5] Guldan H, Sterner R, Babinger P (2008) Identification and characterization of a bacterial glycerol-1-phosphate dehydrogenase: Ni^2+^-dependent AraM from *Bacillus subtilis* . Biochemistry 47: 7376–7384. 18558723 10.1021/bi8005779

[6] Sigel A, Sigel H, Sigel RKO (2007) Nickel and its surprising impact in nature; Sigel A, Sigel H, Sigel RKO, editors. Chichester, West Sussex, England: John Wiley & Sons Ltd. 1912 p.

[7] Forzani C, Lobreaux S, Mari S, Briat JF, Lebrun M (2002) Metal resistance in yeast mediated by the expression of a maize 20S proteasome α subunit. Gene 293: 199–204. 12137958 10.1016/s0378-1119(02)00758-8

[8] Mirete S, de Figueras CG, Gonzalez-Pastor JE (2007) Novel nickel resistance genes from the rhizosphere metagenome of plants adapted to acid mine drainage. Appl Environ Microbiol 73: 6001–6011. 17675438 10.1128/AEM.00048-07 PMC2075024

[9] Macomber L, Hausinger RP (2011) Mechanisms of nickel toxicity in microorganisms. Metallomics 3: 1153–1162. 21799955 10.1039/c1mt00063b PMC4130172

[10] Joho M, Inouhe M, Tohoyama H, Murayama T (1995) Nickel resistance mechanisms in yeasts and other fungi. J Ind Microbiol 14: 164–168. 7766209 10.1007/BF01569899

[11] Nies DH (1999) Microbial heavy-metal resistance. Appl Microbiol Biotechnol 51: 730–750. 10422221 10.1007/s002530051457

[12] Grass G, Grosse C, Nies DH (2000) Regulation of the *cnr* cobalt and nickel resistance determinant from *Ralstonia* sp. strain CH34. J Bacteriol 182: 1390–1398. 10671463 10.1128/jb.182.5.1390-1398.2000 PMC94428

[13] Liesegang H, Lemke K, Siddiqui RA, Schlegel HG (1993) Characterization of the inducible nickel and cobalt resistance determinant *cnr* from pMOL28 of *Alcaligenes eutrophus* CH34. J Bacteriol 175: 767–778. 8380802 10.1128/jb.175.3.767-778.1993 PMC196216

[14] Tibazarwa C, Wuertz S, Mergeay M, Wyns L, van Der Lelie D (2000) Regulation of the *cnr* cobalt and nickel resistance determinant of *Ralstonia eutropha* (*Alcaligenes eutrophus*) CH34. J Bacteriol 182: 1399–1409. 10671464 10.1128/jb.182.5.1399-1409.2000 PMC94429

[15] Stahler FN, Odenbreit S, Haas R, Wilrich J, Van Vliet AH, et al (2006) The novel *Helicobacter pylori* CznABC metal efflux pump is required for cadmium, zinc, and nickel resistance, urease modulation, and gastric colonization. Infect Immun 74: 3845–3852. 16790756 10.1128/IAI.02025-05 PMC1489693

[16] Zhu T, Tian J, Zhang S, Wu N, Fan Y (2011) Identification of the transcriptional regulator NcrB in the nickel resistance determinant of *Leptospirillum ferriphilum* UBK03. PLoS One 6: e17367. 21387010 10.1371/journal.pone.0017367 PMC3046157

[17] Tian J, Wu N, Li J, Liu Y, Guo J, et al (2007) Nickel-resistant determinant from *Leptospirillum ferriphilum* . Appl Environ Microbiol 73: 2364–2368. 17293508 10.1128/AEM.00207-07 PMC1855658

[18] Rawlings DE (2002) Heavy metal mining using microbes. Annu Rev Microbiol 56: 65–91. 12142493 10.1146/annurev.micro.56.012302.161052

[19] Dopson M, Baker-Austin C, Koppineedi PR, Bond PL (2003) Growth in sulfidic mineral environments: metal resistance mechanisms in acidophilic micro-organisms. Microbiology 149: 1959–1970. 12904536 10.1099/mic.0.26296-0

[20] Suzuki K, Wakao N, Kimura T, Sakka K, Ohmiya K (1998) Expression and regulation of the arsenic resistance operon of *Acidiphilium multivorum* AIU 301 plasmid pKW301 in *Escherichia coli* . Appl Environ Microbiol 64: 411–418. 9464374 10.1128/aem.64.2.411-418.1998 PMC106059

[21] Suzuki K, Wakao N, Sakurai Y, Kimura T, Sakka K, et al (1997) Transformation of *Escherichia coli* with a large plasmid of *Acidiphilium multivorum* AIU 301 encoding arsenic resistance. Appl Environ Microbiol 63: 2089–2091. 9143138 10.1128/aem.63.5.2089-2091.1997 PMC168498

[22] San Martin-Uriz P, Gomez MJ, Arcas A, Bargiela R, Amils R (2011) Draft genome sequence of the electricigen *Acidiphilium* sp. strain PM (DSM 24941). J Bacteriol 193: 5585–5586. 21914891 10.1128/JB.05386-11 PMC3187463

[23] Garcia-Moyano A, Gonzalez-Toril E, Aguilera A, Amils R (2012) Comparative microbial ecology study of the sediments and the water column of the Rio Tinto, an extreme acidic environment. FEMS Microbiol Ecol 81: 303–314. 22385317 10.1111/j.1574-6941.2012.01346.x

[24] Gonzalez-Toril E, Llobet-Brossa E, Casamayor EO, Amann R, Amils R (2003) Microbial ecology of an extreme acidic environment, the Tinto River. Appl Environ Microbiol 69: 4853–4865. 12902280 10.1128/AEM.69.8.4853-4865.2003 PMC169134

[25] Malki M, De Lacey AL, Rodriguez N, Amils R, Fernandez VM (2008) Preferential use of an anode as an electron acceptor by an acidophilic bacterium in the presence of oxygen. Appl Environ Microbiol 74: 4472–4476. 18487393 10.1128/AEM.00209-08 PMC2493157

[26] Sanchez-Roman M, Fernandez-Remolar D, Amils R, Sánchez-Navas A, Schmid T, et al.. (2014) Microbial mediated formation of Fe-carbonate minerals under extreme acidic conditions. Sci Rep In press. 10.1038/srep04767 PMC3996482 24755961

[27] Wolin EA, Wolin MJ, Wolfe RS (1963) Formation of Methane by Bacterial Extracts. J Biol Chem 238: 2882–2886. 14063318

[28] Sambrook J, Russell DW (2001) Molecular cloning: a laboratory manual. Cold Spring Harbor, New York: Cold Spring Harbor Laboratory Press.

[29] Altschul SF, Gish W, Miller W, Myers EW, Lipman DJ (1990) Basic Local Alignment Search Tool. Journal of Molecular Biology 215: 403–410. 2231712 10.1016/S0022-2836(05)80360-2

[30] Guazzaroni ME, Morgante V, Mirete S, Gonzalez-Pastor JE (2013) Novel acid resistance genes from the metagenome of the Tinto River, an extremely acidic environment. Environ Microbiol 15: 1088–1102. 23145860 10.1111/1462-2920.12021

[31] Ludwig W, Strunk O, Westram R, Richter L, Meier H, et al (2004) ARB: a software environment for sequence data. Nucleic Acids Res 32: 1363–1371. 14985472 10.1093/nar/gkh293 PMC390282

[32] Pruesse E, Quast C, Knittel K, Fuchs BM, Ludwig W, et al (2007) SILVA: a comprehensive online resource for quality checked and aligned ribosomal RNA sequence data compatible with ARB. Nucleic Acids Res 35: 7188–7196. 17947321 10.1093/nar/gkm864 PMC2175337

[33] Tamura K, Peterson D, Peterson N, Stecher G, Nei M, et al (2011) MEGA5: molecular evolutionary genetics analysis using maximum likelihood, evolutionary distance, and maximum parsimony methods. Mol Biol Evol 28: 2731–2739. 21546353 10.1093/molbev/msr121 PMC3203626

[34] Thompson JD, Higgins DG, Gibson TJ (1994) CLUSTAL W: improving the sensitivity of progressive multiple sequence alignment through sequence weighting, position-specific gap penalties and weight matrix choice. Nucleic Acids Res 22: 4673–4680. 7984417 10.1093/nar/22.22.4673 PMC308517

[35] Dew DW, Muhlbauer R, van Buuren C (1999) Bioleaching of copper sulphide concentrates with mesophiles and thermophiles; Brisbane, Australia.

[36] Rodrigue A, Effantin G, Mandrand-Berthelot MA (2005) Identification of *rcnA* (*yohM*), a nickel and cobalt resistance gene in *Escherichia coli* . J Bacteriol 187: 2912–2916. 15805538 10.1128/JB.187.8.2912-2916.2005 PMC1070376

[37] Park JE, Schlegel HG, Rhie HG, Lee HS (2004) Nucleotide sequence and expression of the *ncr* nickel and cobalt resistance in *Hafnia alvei* 5–5. Int Microbiol 7: 27–34. 15179604

[38] Mahapatra NR, Ghosh S, Deb C, Banerjee PC (2002) Resistance to cadmium and zinc in *Acidiphilium symbioticum* KM2 is plasmid mediated. Curr Microbiol 45: 180–186. 12177739 10.1007/s00284-001-0113-6

[39] Parker CT, Kloser AW, Schnaitman CA, Stein MA, Gottesman S, et al (1992) Role of the *rfaG* and *rfaP* genes in determining the lipopolysaccharide core structure and cell surface properties of *Escherichia coli* K-12. J Bacteriol 174: 2525–2538. 1348243 10.1128/jb.174.8.2525-2538.1992 PMC205891

[40] Parker CT, Pradel E, Schnaitman CA (1992) Identification and sequences of the lipopolysaccharide core biosynthetic genes *rfaQ*, *rfaP*, and *rfaG* of *Escherichia coli* K-12. J Bacteriol 174: 930–934. 1732225 10.1128/jb.174.3.930-934.1992 PMC206172

[41] Sigel RKO, Sigel H (2007) Complex formation of nickel(II) and related metal ions with sugar residues, nucleobases, phosphates, nucleotides, and nucleic acids. In: Sigel A, Sigel H, Sigel RKO, editors. Nickel and its surprising impact in nature. Chichester, West Sussex, England: John Wiley & Sons Ltd. pp. 109–180.

[42] Umbarger HE (1978) Amino acid biosynthesis and its regulation. Annu Rev Biochem 47: 532–606. 354503 10.1146/annurev.bi.47.070178.002533

[43] Santiago B, MacGilvray M, Faustoferri RC, Quivey RG Jr (2012) The branched-chain amino acid aminotransferase encoded by *ilvE* is involved in acid tolerance in *Streptococcus mutans* . J Bacteriol 194: 2010–2019. 22328677 10.1128/JB.06737-11 PMC3318461

[44] Tremaroli V, Workentine ML, Weljie AM, Vogel HJ, Ceri H, et al (2009) Metabolomic investigation of the bacterial response to a metal challenge. Appl Environ Microbiol 75: 719–728. 19047385 10.1128/AEM.01771-08 PMC2632130

[45] Cheng Z, Wei YY, Sung WW, Glick BR, McConkey BJ (2009) Proteomic analysis of the response of the plant growth-promoting bacterium *Pseudomonas putida* UW4 to nickel stress. Proteome Sci 7: 18. 19422705 10.1186/1477-5956-7-18 PMC2689183

[46] Flint DH, Tuminello JF, Emptage MH (1993) The inactivation of Fe-S cluster containing hydro-lyases by superoxide. J Biol Chem 268: 22369–22376. 8226748

[47] Geslin C, Llanos J, Prieur D, Jeanthon C (2001) The manganese and iron superoxide dismutases protect *Escherichia coli* from heavy metal toxicity. Res Microbiol 152: 901–905. 11766965 10.1016/s0923-2508(01)01273-6

[48] Chuang SE, Burland V, Plunkett G III, Daniels DL, Blattner FR (1993) Sequence analysis of four new heat-shock genes constituting the *hslTS/ibpAB* and *hslVU* operons in *Escherichia coli* . Gene 134: 1–6. 8244018 10.1016/0378-1119(93)90167-2

[49] Missiakas D, Schwager F, Betton JM, Georgopoulos C, Raina S (1996) Identification and characterization of HsIV HsIU (ClpQ ClpY) proteins involved in overall proteolysis of misfolded proteins in *Escherichia coli* . EMBO J 15: 6899–6909. 9003766 PMC452516

[50] Rohrwild M, Coux O, Huang HC, Moerschell RP, Yoo SJ, et al (1996) HslV-HslU: A novel ATP-dependent protease complex in *Escherichia coli* related to the eukaryotic proteasome. Proc Natl Acad Sci U S A 93: 5808–5813. 8650174 10.1073/pnas.93.12.5808 PMC39143

[51] Jain R, Chan MK (2007) Support for a potential role of *E. coli* oligopeptidase A in protein degradation. Biochem Biophys Res Commun 359: 486–490. 17553460 10.1016/j.bbrc.2007.05.142

[52] Pruteanu M, Neher SB, Baker TA (2007) Ligand-controlled proteolysis of the *Escherichia coli* transcriptional regulator ZntR. J Bacteriol 189: 3017–3025. 17220226 10.1128/JB.01531-06 PMC1855835

[53] Kanemori M, Nishihara K, Yanagi H, Yura T (1997) Synergistic roles of HslVU and other ATP-dependent proteases in controlling in vivo turnover of σ^32^ and abnormal proteins in *Escherichia coli* . J Bacteriol 179: 7219–7225. 9393683 10.1128/jb.179.23.7219-7225.1997 PMC179669

[54] Lien HY, Yu CH, Liou CM, Wu WF (2009) Regulation of *clpQ^+^Y^+^* (*hslV^+^U^+^*) Gene Expression in *Escherichia coli* . Open Microbiol J 3: 29–39. 19440251 10.2174/1874285800903010029 PMC2681174

[55] Seong IS, Oh JY, Yoo SJ, Seol JH, Chung CH (1999) ATP-dependent degradation of SulA, a cell division inhibitor, by the HslVU protease in *Escherichia coli* . FEBS Lett 456: 211–214. 10452560 10.1016/s0014-5793(99)00935-7

[56] Khattar MM (1997) Overexpression of the *hslVU* operon suppresses SOS-mediated inhibition of cell division in *Escherichia coli* . FEBS Lett 414: 402–404. 9315728 10.1016/s0014-5793(97)01024-7

[57] Goto Y, Calciano LJ, Fink AL (1990) Acid-induced folding of proteins. Proc Natl Acad Sci U S A 87: 573–577. 2153957 10.1073/pnas.87.2.573 PMC53307

[58] Hong W, Jiao W, Hu J, Zhang J, Liu C, et al (2005) Periplasmic protein HdeA exhibits chaperone-like activity exclusively within stomach pH range by transforming into disordered conformation. J Biol Chem 280: 27029–27034. 15911614 10.1074/jbc.M503934200

[59] Richard H, Foster JW (2004) *Escherichia coli* glutamate- and arginine-dependent acid resistance systems increase internal pH and reverse transmembrane potential. J Bacteriol 186: 6032–6041. 15342572 10.1128/JB.186.18.6032-6041.2004 PMC515135

[60] Schonknecht G, Chen WH, Ternes CM, Barbier GG, Shrestha RP, et al (2013) Gene transfer from bacteria and archaea facilitated evolution of an extremophilic eukaryote. Science 339: 1207–1210. 23471408 10.1126/science.1231707

[61] Morgante V, Mirete S, Gonzalez de Figueras C, Postigo Cacho M, Gonzalez-Pastor JE (2014) Exploring the diversity of arsenic resistance genes from acid mine drainage microorganisms. Environ Microbiol: In press. 10.1111/1462-2920.12505 24801164

