# Supplementary material for: Nickel-Resistance Determinants in Acidiphilium sp. PM Identified by Genome-Wide Functional Screening
Source: PLoS One. 2014 Apr 16;9(4):e95041. doi: 10.1371/journal.pone.0095041 (PMC3989265; doi:10.1371/journal.pone.0095041)

**SUPPORTING INFORMATION**

**Nickel-resistance determinants in *Acidiphilium* sp. PM identified by genome-wide functional screening**

Patxi San Martin-Uriz^a^, Salvador Mirete^a^, Pedro J. Alcolea^b^, Manuel J. Gomez^a§^, Ricardo Amils^a,c^, Jose E. Gonzalez-Pastor^a^#

Centro de Astrobiología (INTA-CSIC), Instituto Nacional de Técnica Aeroespacial, Torrejón de Ardoz, Spain^a;^ Centro de Investigaciones Biológicas (CSIC), Consejo Superior de Investigaciones Científicas, Madrid, Spain^b^; Centro de Biología Molecular Severo Ochoa (UAM-CSIC), Universidad Autónoma de Madrid, Madrid, Spain^c^

# Address correspondence to José E. González-Pastor, [gonzalezpje@cab.inta-csic.es](mailto:gonzalezpje@cab.inta-csic.es)

^§^Present address: Manuel J. Gomez, Centro Nacional de Investigaciones Cardiovasculares, Madrid, Spain.

**Figure S1. Determination of heavy-metal cross-resistance of the four Ni-resistant clones.** Serial dilutions of overnight-grown cultures were plated on LB-Ap plates containing 0.8 mM Cd, 1.25 mM Co, 4.5 mM Cu, or 1.5 mM Zn.


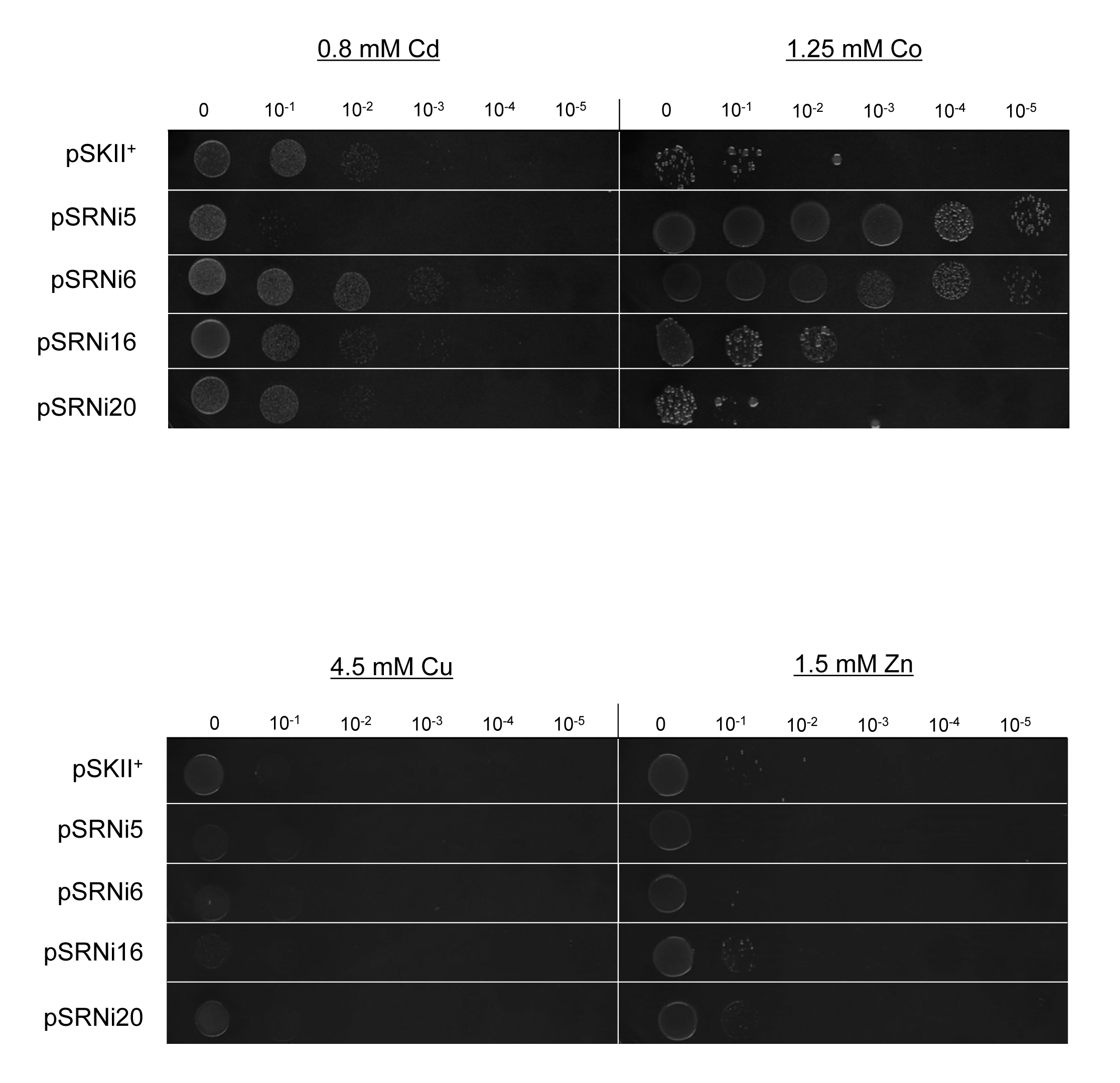


**Figure S2. Identification of the *orfs* involved in Ni resistance by subcloning.** Subclones were constructed and tested for Ni resistance in pSRNi5 (A), pSRNi6 (B) and pSRNi16 (C). Serial dilutions of overnight cultures were plated in LB-Ap plates containing 2.25mM Ni. Asssays were performed in triplicate using independent cultures. pSRNi5_*orf2*: ATP-dependent protease hsIV; pSRNi5_*orf3*: ATP-dependent protease ATP-binding subunit HslU; pSRNi5_*orf5*: amidase; pSRNi6_*orf*1: 3-oxoacyl-(acyl-carrier-protein) reductase; pSRNi6_*orf2*: malonyl CoA-acyl carrier protein transacylase; pSRNi6_*orf3*: polysaccharide export protein; pSRNi6_*orf4*: non-specific protein-tyrosine kinase; pSRNi16_*orf1*: dihydroxy-acid dehydratase; pSRNi16_*orf2*: hypothetical protein; pSRNi16_*orf3*: RND efflux transporter. ORFs involved in Ni resistance are shown in grey. ORFs with predicted transmembrane helices are shaded with vertical bars. Asterisks indicate incomplete ORFs.


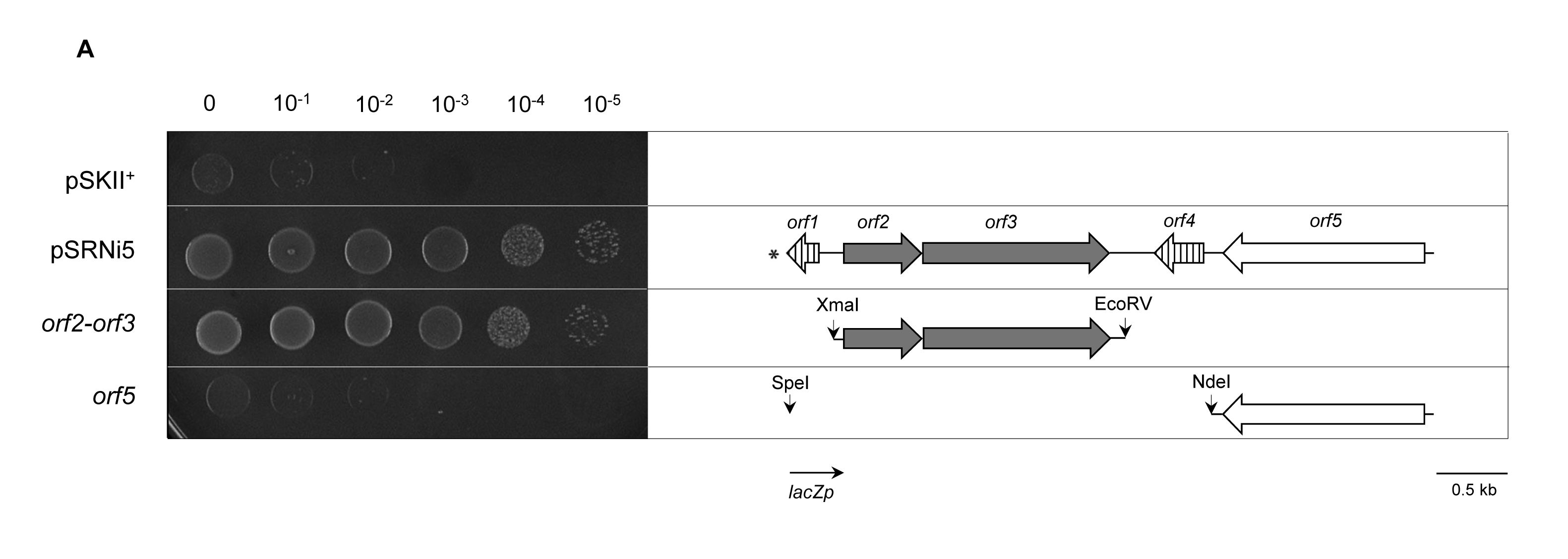


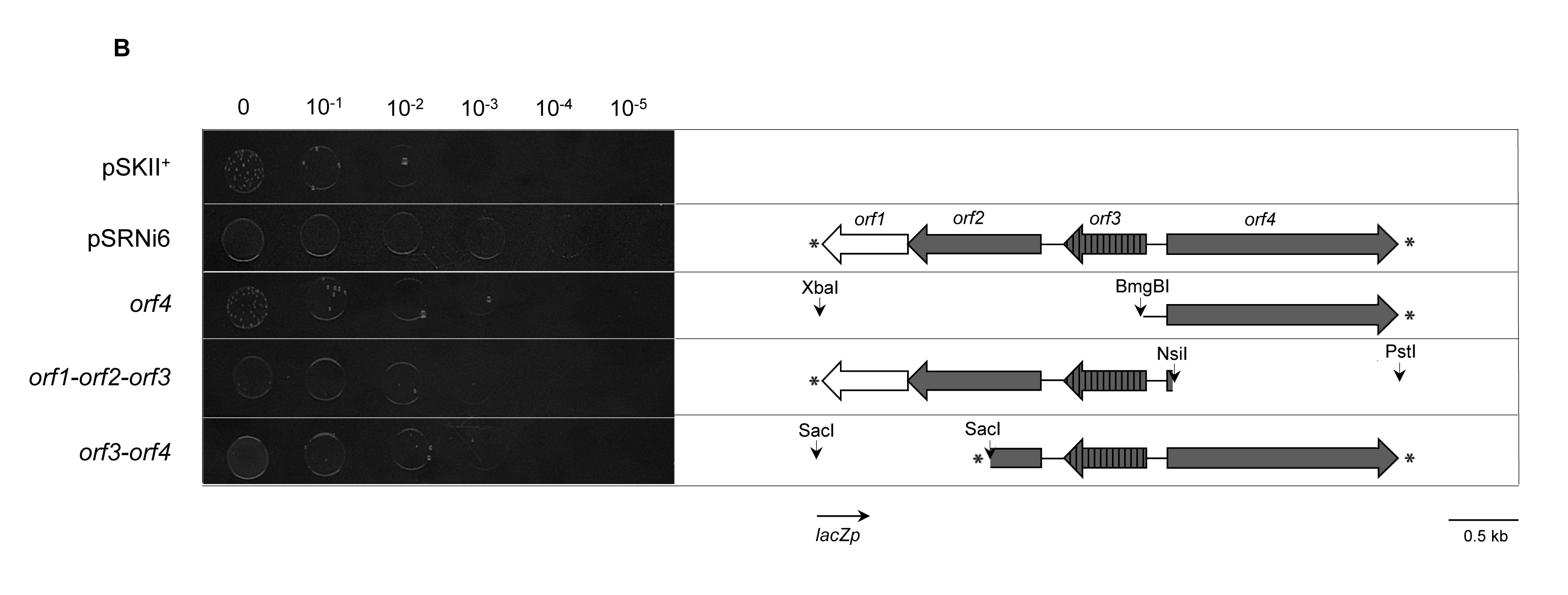


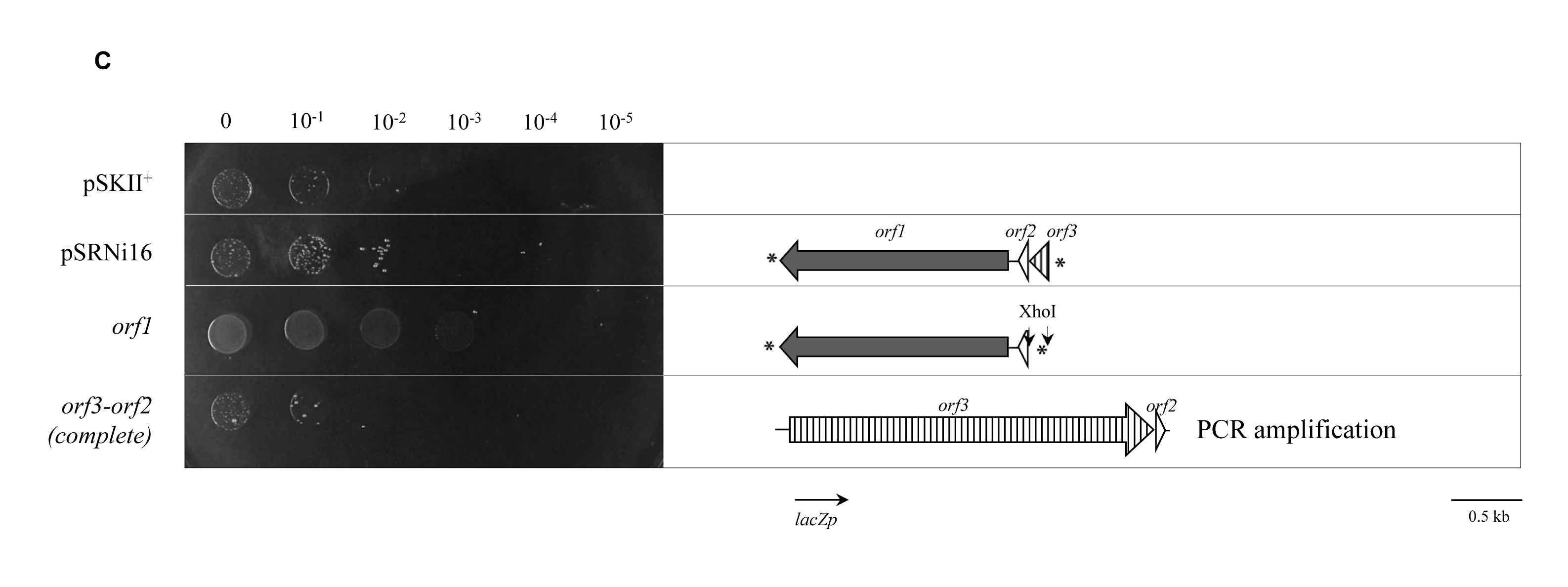


**Figure S3. Determination of heavy-metal cross-resistance of protease HslVU (pSRNi5_*orf2-orf3*).** Serial dilutions of overnight-grown cultures were plated on LB-Ap plates containing 0.8 mM Cd, 1.25 mM Co, 4.5 mM Cu, or 1.5 mM Zn.

**
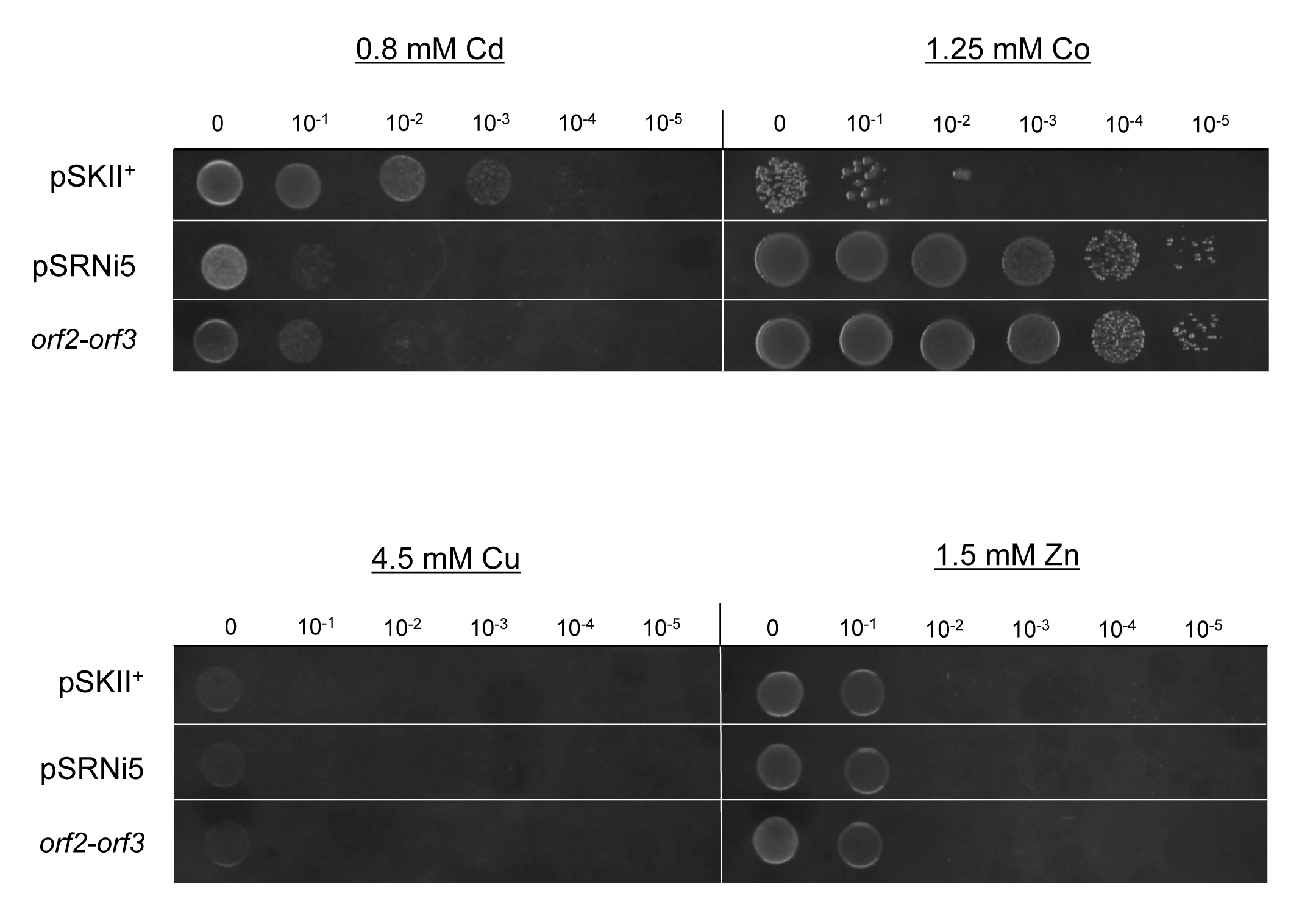
**

**Figure S4. Exploring possible horizontal gene transfer of operon *hslVU* in acidic environments.** Phylogenetic trees of 14 acidic and 14 non-acidic species are shown as reconstructed from the 16S rRNA gene (A), and the concatenated amino acid sequences of HslV and HslU (B). Sequences belonging to *Acidiphilium* sp. PM are shown in bold text and those of acidic species are denoted by asterisks. Bootstrap values are indicated at the nodes. Scale bars correspond to 5% (A) or 10% (B) sequence divergence. The amino acid sequences for HslV and HslU used in the analysis are the following: *Rhodospirillum rubrum* F11, AEO50144 and AEO50145; *Escherichia coli* str. K-12 substr. DH10B, ACB04944 and ACB04943; *Pseudomonas aeruginosa* PAO1, AAG08438.1 and AAG08439; *Bacillus subtilis* subsp. *subtilis* str. 168, CAB13488 and CAB13489; *Salinibacter ruber* M8, CBH24765 and CBH24767; *Ralstonia solanacearum* GMI1000, CAD13571 and CAD13570; *Borrelia burgdorferi* N40, ADQ29063 and ADQ29174; *Thermotoga maritima* MSB8, AAD35606 and AAD35607; *Planctomyces brasiliensis* DSM 5305, ADY60041 and ADY60040; *Acidithiobacillus ferrooxidans* ATCC 23270, ACK80450 and ACK79019; *Acidithiobacillus ferrooxidans* ATCC 53993, ACH84568 and ACH84569; *Acidithiobacillus ferrivorans* SS3, AEM46768 and AEM46767; *Acidithiobacillus caldus* SM-1, AEK57130 and AEK57129; *Acidithiobacillus caldus* ATCC 51756, EET27426 and EET27425; *Acidiphilium multivorum* AIU301, BAJ80804 and BAJ80803; *Acidiphilium* sp. PM, EGO96503 and EGO96504; *Acidiphilium cryptum* JF-5, ABQ30620 and ABQ30619; *Leptospirillum rubarum*, EAY57767 and EAY57766; *Leptospirillum* sp. Group II '5-way CG', EDZ39580 and EDZ39579; *Acidocella* sp. MX-AZ02, EKM99984 and EKM99983; *Acetobacter pasteurianus* IFO 3283-01, BAH99172 and BAH99173; *Gluconacetobacter diazotrophicus* PAl 5, ACI52812 and ACI52813; *Gluconobacter oxydans* H24, AFW01689 and AFW01690; *Roseomonas cervicalis* ATCC 49957, EFH12145 and EFH12146; *Azospirillum lipoferum* 4B, CBS88212 and CBS88211; *Thiomonas intermedia* K12, ADG31586 and ADG31587; *Sulfobacillus acidophilus* DSM 10332, AEW05868 and AEW05867; *Alicyclobacillus acidocaldarius* subsp. *acidocaldarius* DSM 446, ACV58411 and ACV58412; *Leptothrix cholodnii* SP-6, ACB36091 and ACB36092.
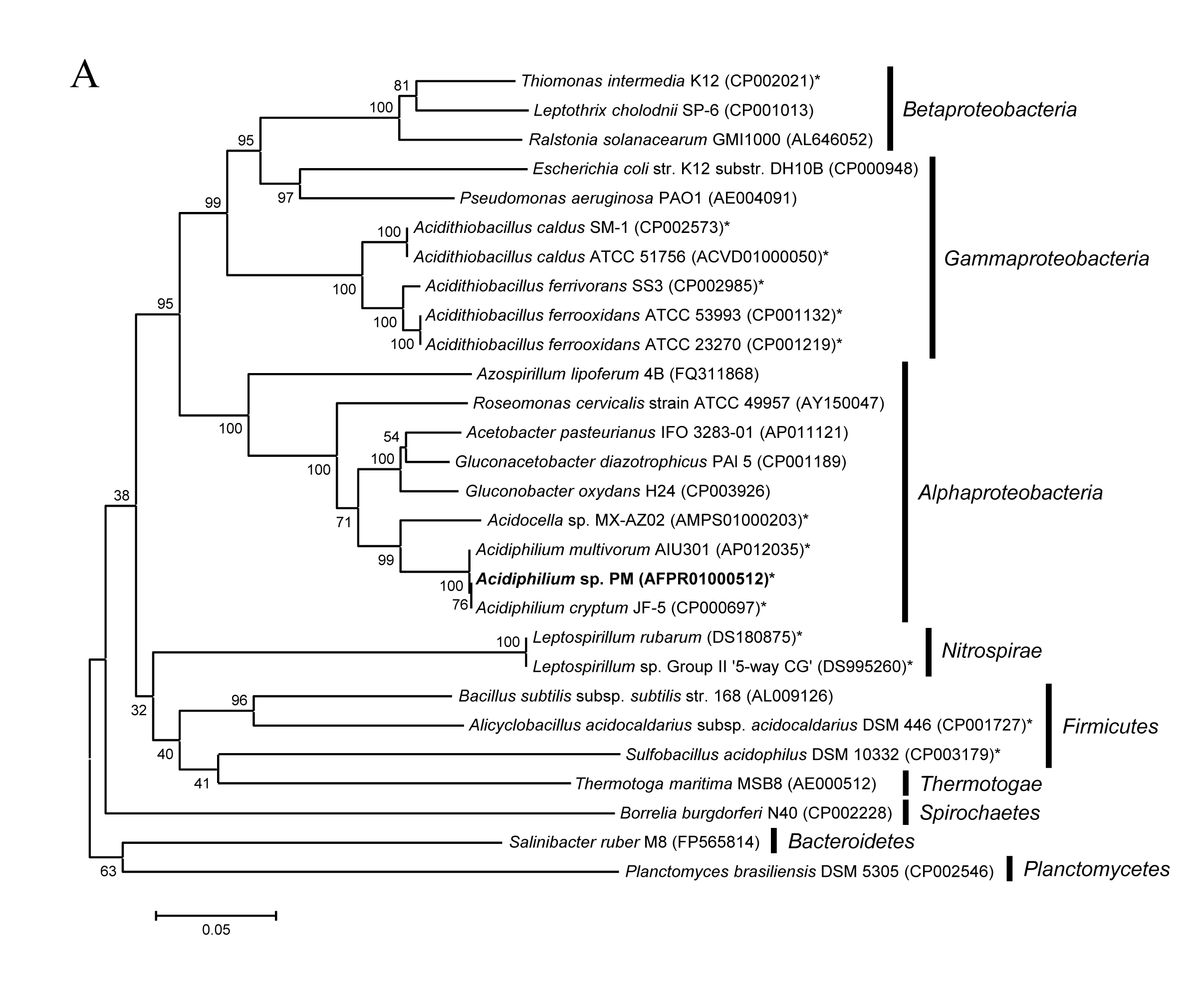


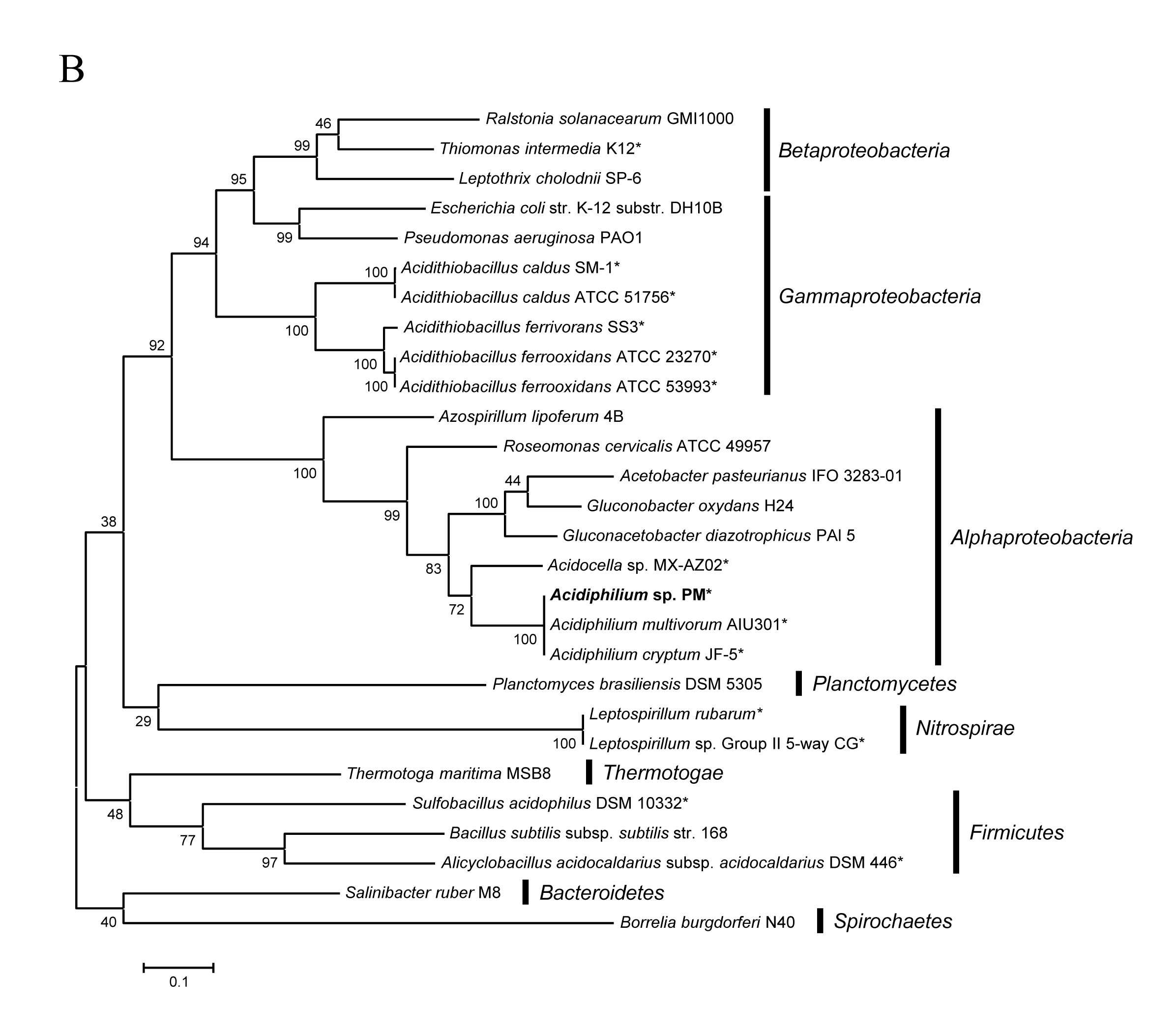

Supplement: File S1 — Figure S1. Determination of heavy-metal cross-resistance of the four nickel-resistant clones. Serial dilutions of overnight-grown cultures were plated on LB-Ap plates containing 0.8 mM Cd, 1.25 mM Co, 4.5 mM Cu, or 1.5 mM Zn. Figure S2. Identification of the orfs involved in Ni resistance by subcloning. ORFs from the environmental DNA inserts of pSRNi5 (A), pSRNi6 (B) and pSRNi16 (C) were subcloned and tested for Ni resistance. Serial dilutions of overnight cultures were plated in LB-Ap plates containing 2.25 mM Ni. Assays were performed in triplicate using independent cultures. ORFs involved in Ni resistance are shown in grey. ORFs with predicted transmembrane helices are shaded with vertical bars. Asterisks indicate incomplete ORFs. pSRNi5_orf2: ATP-dependent protease hsIV; pSRNi5_orf3: ATP-dependent protease ATP-binding subunit HslU; pSRNi5_orf5: amidase; pSRNi6_orf1: 3-oxoacyl-(acyl-carrier-protein) reductase; pSRNi6_orf2: malonyl CoA-acyl carrier protein transacylase; pSRNi6_orf3: polysaccharide export protein; pSRNi6_orf4: non-specific protein-tyrosine kinase; pSRNi16_orf1: dihydroxy-acid dehydratase; pSRNi16_orf2: hypothetical protein; pSRNi16_orf3: RND efflux transporter. Figure S3. Determination of heavy-metal cross-resistance of protease HslVU (orf2-orf3). Serial dilutions of overnight-grown cultures were plated on LB-Ap plates containing 0.8 mM Cd, 1.25 mM Co, 4.5 mM Cu, or 1.5 mM Zn. Figure S4. Exploring possible horizontal gene transfer of operon hslVU in acidic environments. Phylogenetic trees of 14 acidic and 14 non-acidic species are shown as reconstructed from the 16S rRNA gene (A), and the concatenated amino acid sequences of HslV and HslU (B). Sequences belonging to Acidiphilium sp. PM are shown in bold text and those of acidic species are denoted by asterisks. Bootstrap values are indicated at the nodes. Scale bars correspond to 5% (A) or 10% (B) sequence divergence. The amino acid sequences for HslV and HslU used in the analysis are the followin [file pone.0095041.s001.docx]
